# Glyco-Coated CdSe/ZnS Quantum Dots as Nanoprobes for Carbonic Anhydrase IX Imaging in Cancer Cells

**DOI:** 10.1021/acsanm.1c03603

**Published:** 2021-11-17

**Authors:** Giacomo Biagiotti, Andrea Angeli, Arianna Giacomini, Gianluca Toniolo, Luca Landini, Gianluca Salerno, Lorenzo Di Cesare Mannelli, Carla Ghelardini, Tommaso Mello, Silvia Mussi, Cosetta Ravelli, Marcello Marelli, Stefano Cicchi, Enzo Menna, Roberto Ronca, Claudiu T. Supuran, Barbara Richichi

**Affiliations:** † Department of Chemistry “Ugo Schiff”, University of Firenze, Via della Lastruccia 13, Sesto Fiorentino, 50019 Florence, Italy; ‡ Consorzio Interuniversitario Nazionale per la Scienza e Tecnologia dei Materiali (INSTM, Via G. Giusti, 9, 50121 Firenze, Florence, Italy; § Department of Neuroscience, Psychology, Drug Research and Child Health − NEUROFARBA, Section of Pharmaceutical Chemistry, University of Firenze, Via Ugo Schiff 7, Sesto Fiorentino, 50019 Florence, Italy; ∥ Department of Molecular and translational Medicine, University of Brescia, Viale Europa 11, 25123 Brescia, Italy; ⊥ Department of Neuroscience, Psychology, Drug Research and Child Health - NEUROFARBA - Pharmacology and Toxicology Section, University of Firenze, V.le Pieraccini 6, 50139 Firenze, Florence, Italy; # Department of Clinical and Experimental Biomedical Sciences “Mario Serio”—Gastroenterology Unit, University of Firenze, V.le Pieraccini 6, 50139 Firenze, Florence, Italy; ∇ Istituto di scienze e tecnologie chimiche “Giulio Natta”, CNR-SCITEC, Sede Fantoli, Via Fantoli 16/15, 20138 Milano Italy; ○ Department of Chemical Sciences, University of Padova, Via Marzolo 1, 35131 Padova, Italy; ◆ Centre for Mechanics of Biological Materials—CMBM, Via Marzolo 9, 35131 Padova, Italy

**Keywords:** glyco-quantum dots, carbonic anhydrase, bioimaging, bladder cancer, sulfonamide

## Abstract

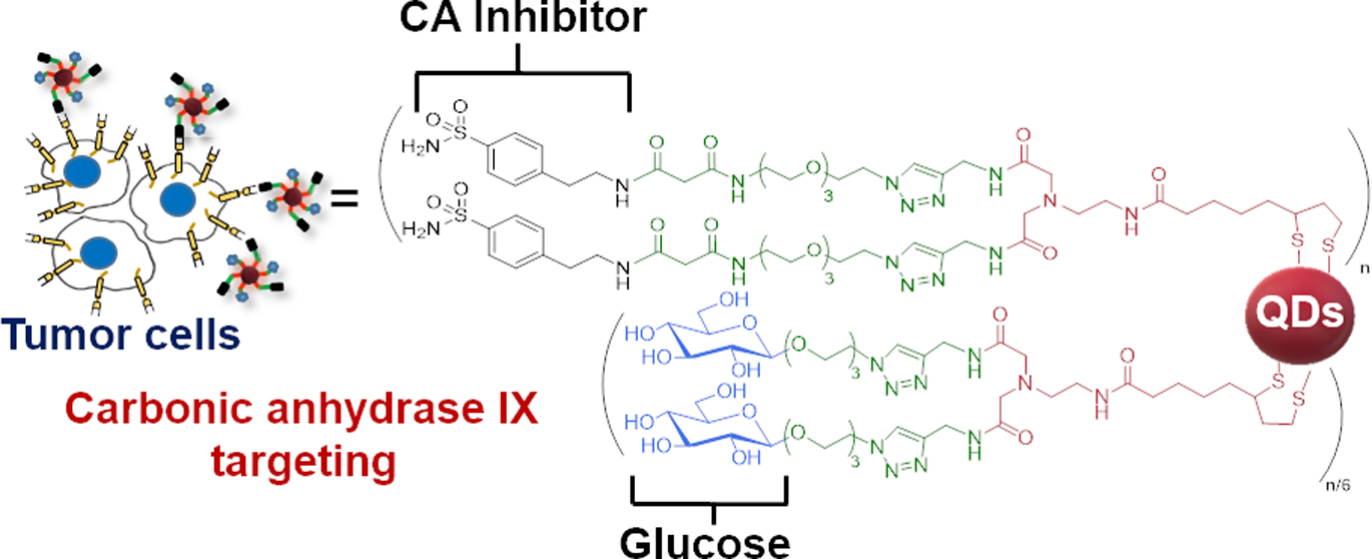

The bioimaging of cancer cells by the specific targeting of overexpressed biomarkers is an approach that holds great promise in the identification of selective diagnostic tools. Tumor-associated human carbonic anhydrase (hCA) isoforms IX and XII have been considered so far as well-defined biomarkers, with their expression correlating with cancer progression and aggressiveness. Therefore, the availability of highly performant fluorescent tools tailored for their targeting and able to efficiently visualize such key targets is in high demand. We report here on the design and synthesis of a kind of quantum dot (QD)-based fluorescent glyconanoprobe coated with a binary mixture of ligands, which, according to the structure of the terminal domains, impart specific property sets to the fluorescent probe. Specifically, monosaccharide residues ensured the dispersibility in the biological medium, CA inhibitor residues provided specific targeting of membrane-anchored hCA IX overexpressed on bladder cancer cells, and the quantum dots imparted the optical/fluorescence properties.

## Introduction

Salient features, such as an hypoxic environment and acidosis, differentiate tumor cells from normal cells and are the result of a complex molecular machinery that promotes overexpression of proteins involved in pH regulation (carbonic anhydrases, sodium-proton exchangers, sodium-bicarbonate cotransporters, to mention some of them), glucose metabolism (glucose transporters, lactate dehydrogenase, etc.), and angiogenesis (VEGF).^1−4^ Targeting some of these proteins has been hypothesized and then demonstrated to constitute innovative approaches for antitumor/antimetastatic therapies, with several drugs already in clinical use or in late stages of clinical development.^4−7^ In this context, inhibitors of the tumor-associated carbonic anhydrase (CA) isoforms IX and XII, such as SLC-0111, represent successful approaches to target the differential pH regulation and metabolism of tumor cells and are actually in phase Ib/II clinical trials.^1,7,8^

Indeed, CA IX and XII, members of the superfamily of α-CAs, zinc enzymes that catalyze the hydration of CO_2_ to bicarbonate and protons,^9^ are significantly overexpressed in many tumors while being present in few normal tissues at rather low expression levels, which makes them excellent drug targets.^10^ As a consequence, a variety of small-molecule CA inhibitors (CAIs) belonging to many diverse chemical classes,^8−10^ small-molecule drug conjugates (SMDCs),^10^ antibody–drug conjugates (ADACs),^11^ or cytokine–drug conjugates targeting CA IX/XII^12^ have been proposed over the last decade and showed significant antitumor activity.^8^ Nanoparticles decorated with CAIs of the sulphonamide type also showed promising *in vitro* antiproliferative action.^13^ In addition, fluorescent CA IX inhibitors have been developed for tumor bioimaging^14^ and showed relevant properties in the experiments that validated CA IX/XII as drug targets.^15^ Although several types of different fluorescent moieties have been attached to CA IX/XII inhibitors to investigate their distribution, membrane localization, and properties,^16−18^ quantum dots (QDs) involving these enzymes have not been used for these applications so far.

Luminescent QDs are well-known nanocrystals that have emerged as highly performant and versatile nanotools.^19,20^ They have been intensively studied and diverse modifications mainly in terms of surface manipulation and preparation of high-quality semiconductor nanocrystals have been proposed thus allowing to exploit QDs (among many others) for diverse *in vitro* and *in vivo* applications including imaging, sensing, and diagnosis.^21−24^

**Figure 1:**
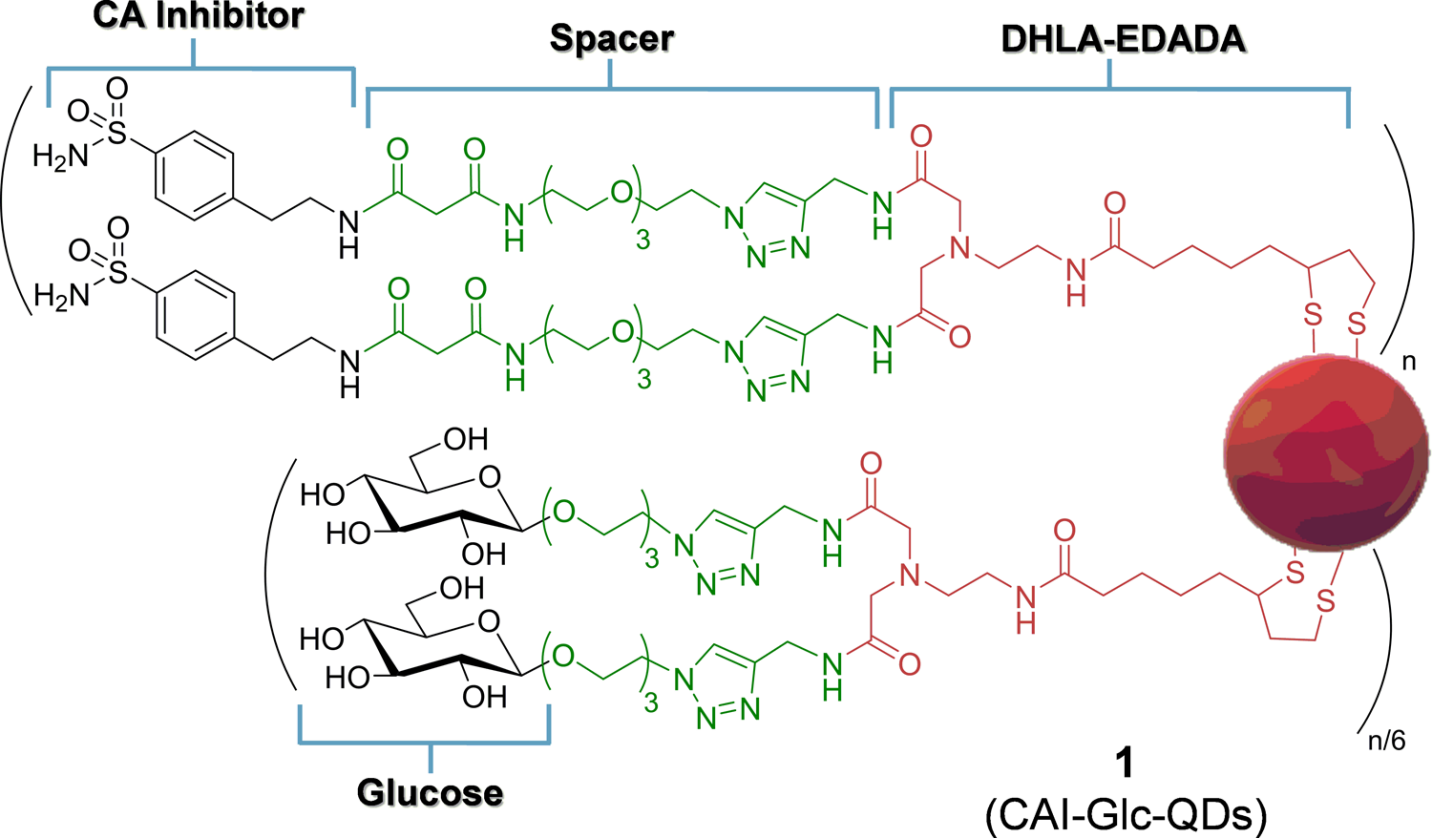
Schematic representation of the core–shell CdSe/ZnS QDs (nanoprobe **1**, CAI-Glc-QDs) coated with a binary mixture of ligands, which contain carbonic anhydrase inhibitor (CAI) and d-glucose (Glc) residues as terminal domains.

In this framework, we have recently demonstrated that a small heterobifunctional ligand named DHLA-EDADA^24,25^ was able to provide highly fluorescent CdSe/ZnS QD suspensions that showed remarkable colloidal stability. Indeed, DHLA-EDADA coated QDs were stable over extended periods of time and over a wide pH range and with different buffer types (*i.e*. PBS, TRIS, DMEM). The DHLA-EDADA ligand (Figure 1) consisted of a dihydrolipoic acid (DHLA) residue, which provides thiol groups with high affinity for the ZnS shell, which is conjugated to the terminal amine group of an ethylenediamine-*N*,*N*-diacetic acid residue (EDADA), which, in turn, provides two-terminal carboxylic groups suitable for further conjugations.^25^

On this basis, we decided to exploit the remarkable colloidal stability provided to QD suspensions by the presence of the DHLA-EDADA ligand on their surface and to prepare the CAI-grafted fluorescent nanoprobe **1** (CAI-Glc-QDs; Figure 1). In particular, we report here on the synthesis of the nanoprobe **1** (Figure 1) and its use to imaging bladder cancer cells by the targeting of overexpressed membrane-anchored CA IX.

Nanoprobe **1** consists of CdSe/ZnS QDs coated with a binary mixture of ligands, which, according to the structure of the terminal domains, impart the specific property sets to the fluorescent probe. The two ligand shells, assembled in a roughly 6:1 ratio on the QDs surface, contain 4-aminoethylbenzene sulphonamide, a well-known CAI,^9^ and d-glucose (Glc) residues, ensuring each cancer-targeting ability and water dispersibility to the nanoprobe **1**. Such terminal domains are oriented toward the surrounding medium and they are both conjugated, through a poly(ethylene glycol) (PEG) spacer differing in length, to a DHLA-EDADA molecule. The mixed-ligand nanoparticles have been assayed in a stopped-flow assay *vs* a panel of CAs and they show interesting inhibition. *In vitro* confocal bioimaging on bladder cancer cells offers a proof of concept of the ability of glyco-QDs probe **1** to target and decorate cancer cells by the specific recognition of tumor-associated membrane-anchored hCA IX.

## Results and Discussion

### Synthesis of CAI-Glc-QD Nanoprobe 1

**Scheme 1:**
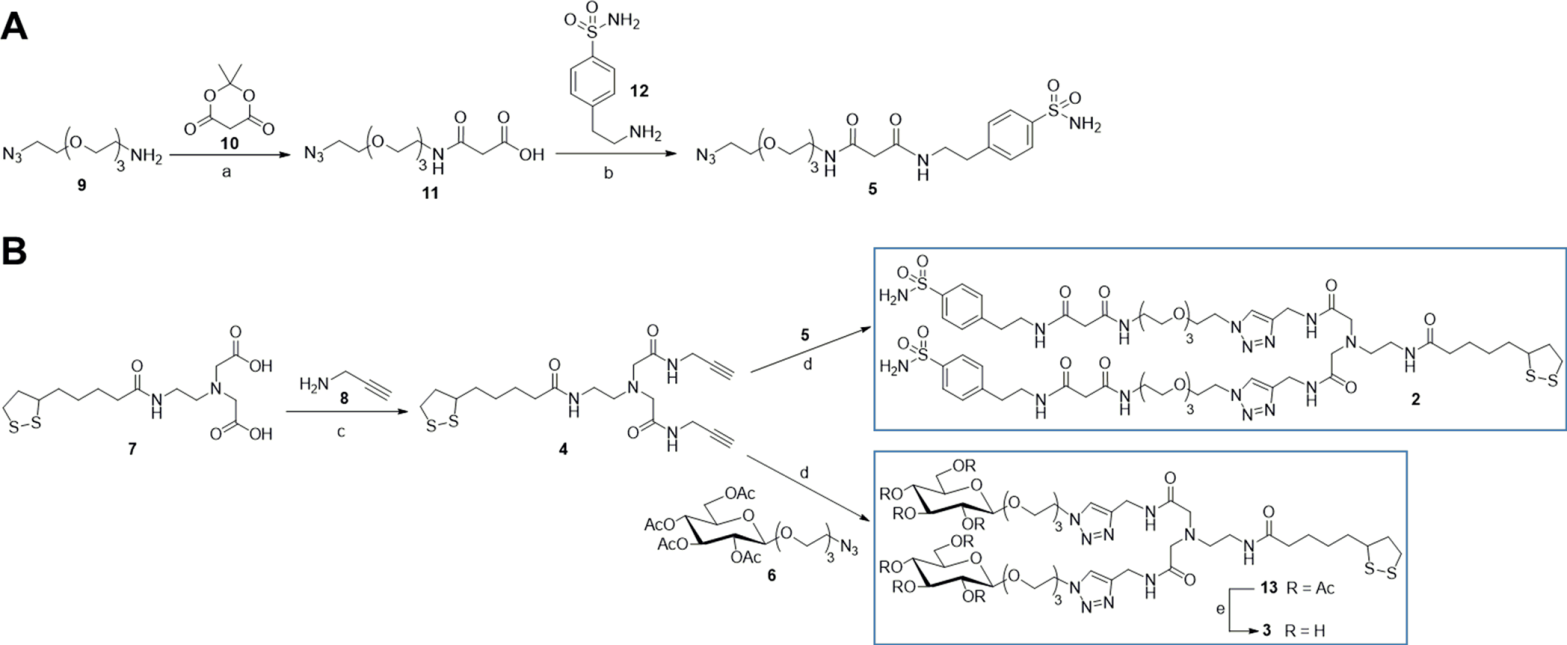
Synthetic Strategy Employed for the Preparation of Ligand Shells **2** and **3** (**A**) Synthesis of compound **5**. Reaction conditions: (a) DCM, 40 °C, 18 h, 96% yield; (b) carbonyl diimidazole (CDI), *N*-methyl morpholine (NMM), DMF, 10 min at 0 °C, then 2 h r.t., 75% yield. (**B**) Synthesis of the ligand shells **2** and **3**. Reaction conditions: (c) TBTU, NMM, DMF, 0 °C to r.t., 12 h, 75% yield; (d) CuSO_4_, sodium ascorbate, DMF, 4.5 h, 69% yield for **2** and 62% yield for **13**; and (e) K_2_CO_3_, MeOH, 24 h r.t., 57% yield.

Several attempts have been made before defining the structure and the composition of the nanoprobe for the CA IX imaging. The main issue was related to the dispersibility of the resulting surface engineered QDs that was significantly affected by the type and composition of the ligand shells (data not shown). In particular, PEG spacers of different lengths between the CAI residue and the terminal carboxylic groups of the DHLA-EDADA residue were introduced. However, any attempt to recover the QDs from the ligand-exchange steps was not successful due to problems related to the dispersibility of the final QDs. Recent reports^24,26−28^ support the use of monosaccharide derivatives on the nanoparticle surface to provide biocompatibility and colloidal stability to the resulting glyco-coated nanoparticles. Thus, the introduction of glucose (Glc) residues combined with the CAI residues on the QDs surface was planned and the final structures of the divalent CAI- and Glc-bearing ligand shells (compounds **2** and **3**) are reported in Scheme 1.

**Figure 2:**
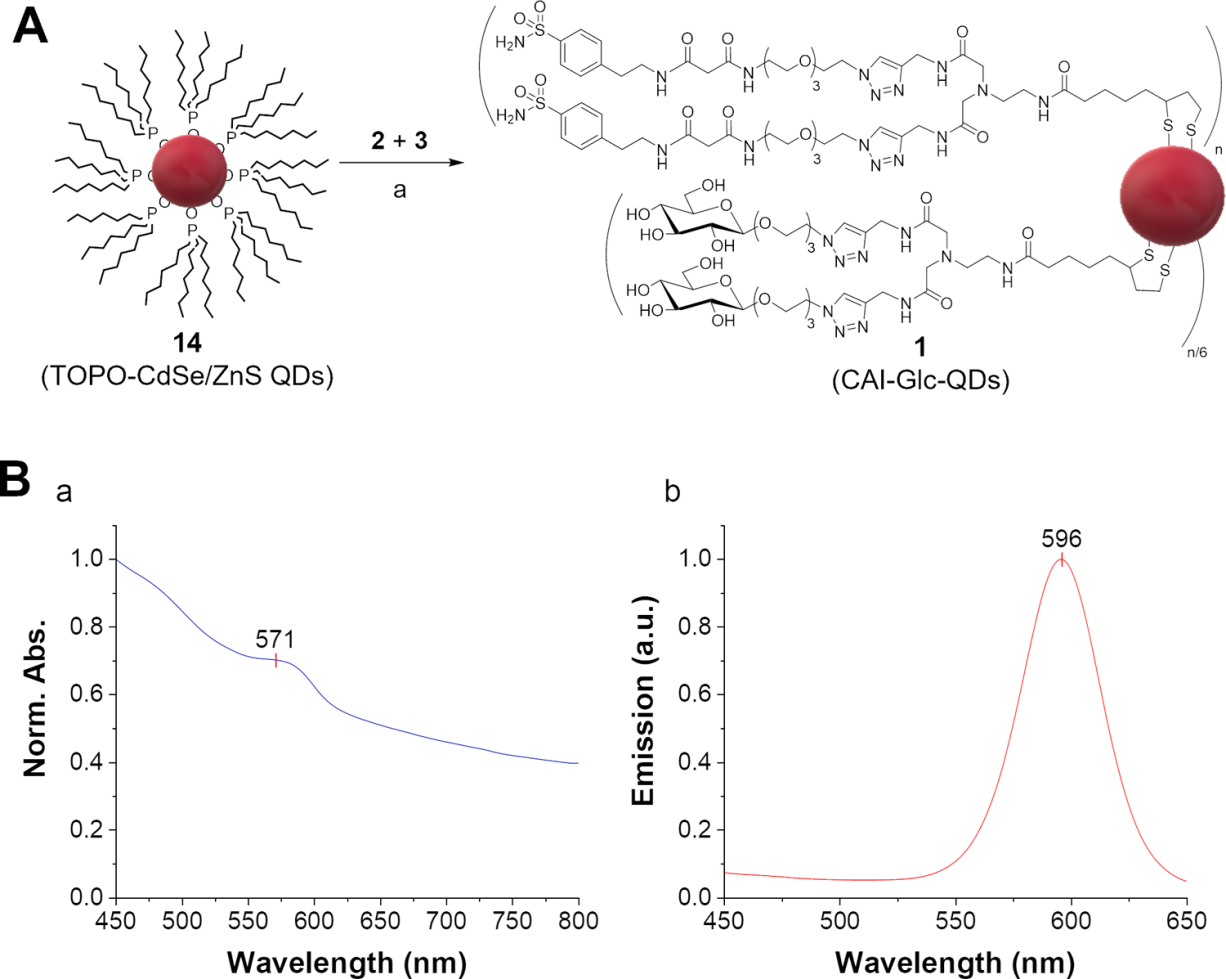
(**A**) Synthesis of CAI-Glc-grafted CsSe/ZnS QDs **1**. Reaction conditions: (a) **2**:**3** (1:2 ratio) in CHCl_3_/H_2_O/MeOH (3:2:1 ratio), NaBH_4_, 40 min, r.t. (**B**) (a) Absorbance spectrum of CAI-Glc-QDs **1** in DMSO and (b) emission spectrum (λ_exc_ = 405 nm) of CAI-Glc-QDs **1** in DMSO.

They were prepared by following a synthetic strategy that includes a key copper(I)-catalyzed azide-alkyne cycloaddition reaction (CuAAC) between the terminal alkyne residues of the DHLA-EDADA derivative **4** and the terminal azide residues of the sulfonamide derivative **5** (Scheme 1A) and the acetylated β-O-glucoside **6**,^29^ respectively (Scheme 1B). The bifunctional di-alkyne derivative **4** was easily prepared in high yield (75% yield) by 2-(1*H*-benzotriazole-1-yl)-1,1,3,3-tetramethylaminium tetrafluoroborate (TBTU) mediated coupling of DHLA-EDADA **7**(25) with the commercially available propargylamine **8** (Scheme 1B). In turn, the synthesis of **5** (Scheme 1A) was performed by the acylation chemistry of the PEGylated amine **9**(30) with the commercially available Meldrum’s acid **10** in mild conditions (40 °C, 18 h, DCM, 96% yield). Thereafter, the carboxylic group of the intermediate derivative **11** (Scheme 1A) was coupled with the commercially available 4-aminoethylbenzene sulfonamide **12** upon reaction with carbonyl diimidazole (CDI) to afford compound **5** in good yield (75% yield). Then, the tetracetate β-O-glucoside **6** (Scheme 1B), bearing a PEGylated linker at the anomeric position, was prepared by modifying a previously reported protocol (Scheme S1, Supporting Information).^29^ Finally, the CuAACs were accomplished using a combination of copper(II) sulfate (CuSO_4_) and sodium ascorbate in dry dimethylformamide (DMF) to afford ligands **2** and **13** in good yields (69% **2** and 62% **13**; Scheme 1B). The glucose residues of **13** were deacetylated under basic conditions (K_2_CO_3_, MeOH, 24 h, 57% yield) affording the divalent Glc-bearing ligand **3**. Trioctylphosphine oxide (TOPO)-coated CdSe/ZnS nanocrystals **14** (Figure 2A and Scheme S2, Supporting Information) were prepared, as previously reported.^25^ Then, nanoprobe **1** (Figure 2A) was prepared by exploiting the well-known surface exchange reaction on the TOPO-coated CdSe/ZnS QDs **14** and using a mixture of thiolate ligands **2** and **3**.

Indeed, as previously reported by some of us,^25^ the reductive opening of the disulfide bridge in the DHLA domains of **2** and **3** (NaBH_4_, CHCl_3_/H_2_O/MeOH (3:2:1 ratio)) produced the bidentate thiol groups, which quickly reacted with the zinc shell of lipophilic QDs **14** (Figure 2A). The corresponding CAI-Glc-QDs **1** were purified by centrifugation (6000 rpm, 5 min) and then nanocrystals were fully characterized (Figures 2B and S1–S4, Supporting Information). In particular, the fluorescence spectrum of the nanoprobe **1** was collected using an excitation wavelength of 405 nm (Figure 2B), and it showed a narrow emission band at λ_em_ = 596 nm. As a result of transmission electron microscopy (TEM) analysis, CAI-Glc-QDs **1** appeared as roundish NPs slightly elongated, ranging in size from 3 to 6 nm (Figure S1I, Supporting Information), as expected from fluorescence emission spectrum (Figure 2B). High-resolution TEM (HRTEM) analysis (Figure S1II–IV, Supporting Information) highlights the high crystallinity and the anisotropic morphology, whereas FFT image analysis confirms the presence of CdSe and ZnS nanocrystals (Figure S2, Supporting Information).

Then, the ligand-shell composition was investigated by nuclear magnetic resonance (NMR) spectroscopy (Figures S3 and S4, Supporting Information) and thermogravimetric analysis (TGA) (Figure S5, Supporting Information).^31^ In the 1D NMR experiments, acetonitrile was used as internal standards (IS), since it displays a ^1^H-NMR signal at a chemical shift value (2.05 ppm), which is free of other ^1^H-NMR signals related to the ligands grafted on CAI-Glc-QDs **1**. The analysis of the 1D ^1^H spectrum in DMSO-*d*6 of QDs **1** (Figure S3, Supporting Information) showed the presence of broad peaks, as expected from ligands linked to the nanoparticle shell. Then, ^1^H-NMR data of QDs **1** (Figure S4B, Supporting Information) showed characteristic signals (7.28 and 7.36 ppm) of the aromatic hydrogens of the aryl sulfonamide moiety of ligand **2** (Figure S4C, Supporting Information), whereas the broad signal observed at 2.76 ppm was attributed to the hydrogens of the methylene group directly linked to the aromatic ring of ligand **2** (Figure S4C, Supporting Information). The presence of the glucose-bearing ligand **3** was confirmed by the anomeric hydrogen peak of the Glc moiety at 4.12 ppm (Figure S4A, Supporting Information). Then, we calculated (*e.g*., peak integration) that about 2.9 × 10^–1^ μmol/mg of **2** and 5.2 × 10^–2^ μmol/mg of **3** have been loaded onto the QD surface, corresponding to 47% of the total weight (Figure S4B, Supporting Information). The results from NMR were consistent with the results obtained by TGA (Table S1, Supporting information).^32^ Indeed, the TGA trace of QDs **1** (Figure S5, Supporting Information) shows a mass loss of 45% of the total weight in the range of temperature corresponding to the decomposition of ligands (Table S1, Supporting information).^31^

### Carbonic Anhydrases Inhibition Assay

The CAI-Glc-QDs **1** were evaluated for their inhibition against the hCA isoforms of interest. Thus, QDs **1** and the ligands **2** and **3** were tested *in vitro* for their inhibitory activity against the human CA isoforms IX, XII, and the off-target hCA I, II by means of a previously reported stopped-flow carbon dioxide hydration assay.^33^ Their activities were compared to those of the well-known CA inhibitor acetazolamide (**AAZ**) and are shown in Tables 1 and 2. Initially, the two ligands **2** and **3** were evaluated separately to understand their inhibition profile against the CA isoforms mentioned above. Data obtained confirmed that, as expected, ligand **2** inhibits both membrane-associated hCA isoforms IX and XII, with a higher selectivity toward hCA XII (Table 1).

**Table 1:** Inhibition of Human CA Isoforms I, II, IX, and XII Using Ligands **2**, **3**, and **AAZ** (Stopped-Flow CO_2_ Hydrase Assay).^33^

K_I_ nM^a^				
Compounds	hCA I	hCAII	hCA IX	hCA XII
**2**	179.8	317.9	415.0	18.1
**3**	>10 000	>10 000	>10 000	>10 000
**AAZ**	250.0	12.1	25.8	5.7

a Mean from three different assays, by a stopped-flow technique (errors were in the range of ±5–10% of the reported values).

**Table 2:** Inhibition of Human CA Isoforms I, II, IX, and XII Using CAI-Glc-QDs **1** and AAZ (Stopped-Flow CO_2_ Hydrase Assay).^33^

K_I_ (mg/ml)^a^				
Compound	hCA I	hCAII	hCA IX	hCA XII
**QDs 1**	9.6 × 10^–4^	5.3 × 10^–4^	6.7 × 10^–4^	3.5 × 10^–5^

a Mean from three different assays, by a stopped-flow technique (errors were in the range of ±5–10% of the reported values).

As expected, for glucose-bearing ligand **3**, we did not observe any activity against the four isoforms, proving that only the CAI-bearing ligand **2** modulates CA activity. Subsequently, we studied the inhibition profile of CAI-Glc-QDs **1**, thus confirming its ability to bind and modulate the activity of the membrane-associated hCAs (hCA IX and hCA XII; Table 2).

### *In Vitro* Confocal Bioimaging Analysis

*In vitro* studies were performed to validate the capacity of the nanoprobe **1** to selectively target CA IX expressed on the cell surface of cancer cells.

For this purpose, the RT4 bladder cancer cells, that express high levels of membrane-anchored CA IX, were cultured under hypoxic conditions and the overexpression of CA IX was confirmed by immunocytochemistry (Figure S6A, Supporting Information) and Western blot analysis (Figure S6C, Supporting Information) using a specific M75 anti-CA IX antibody. To exclude any toxic effect exerted by ligands **2** and **3**, RT4 cells were treated with increasing concentrations of both compounds and no significant effect was observed on cells proliferation up to 100 μM of concentration (Figure S7, Supporting Information).

**Figure 3:**
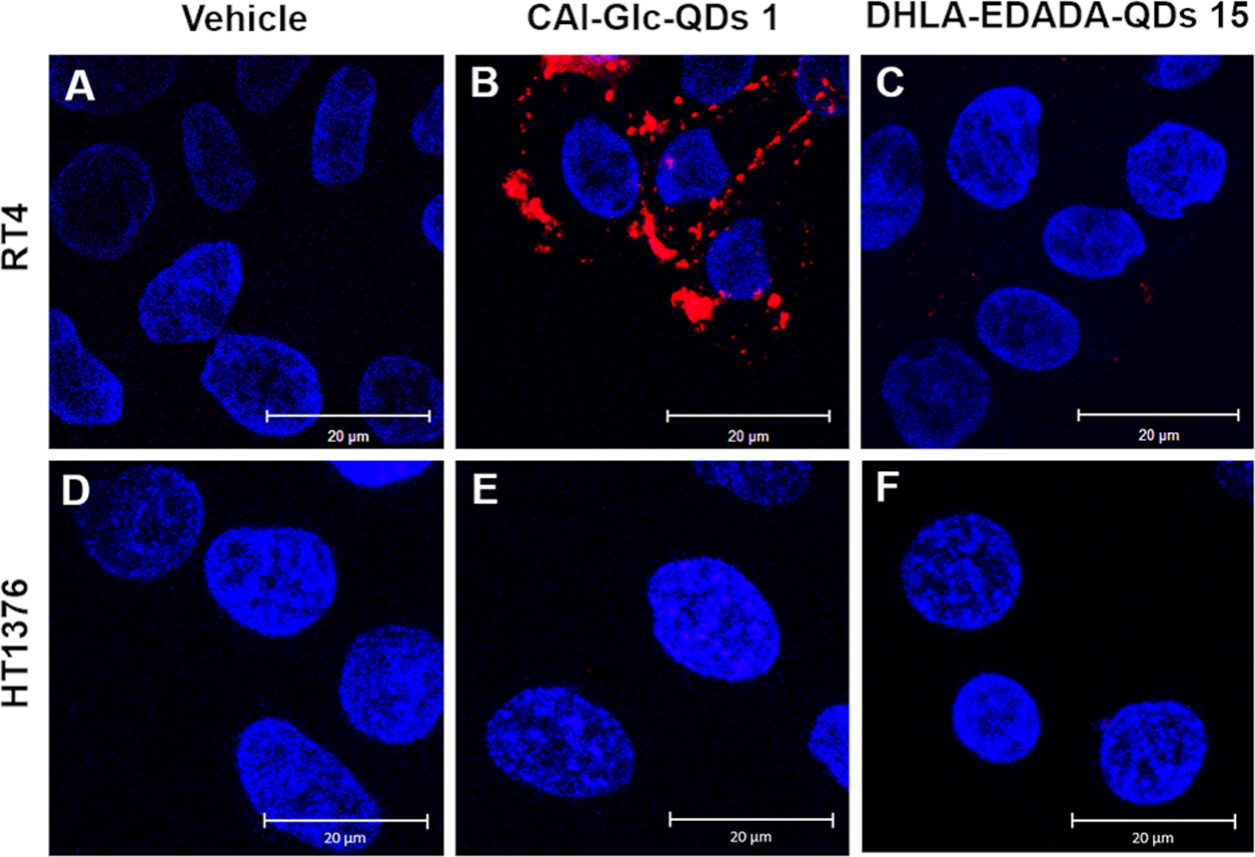
*In vitro* confocal microscopy imaging of RT4 (**A**–**C**) and HT1376 (**D**–**F**) bladder cancer cells incubated with vehicle (**A**, **D**), CAI-Glc-QDs **1** (**B**, **E**), and control DHLA-EDADA-QDs **15** (**C**, **F**). QD fluorescence in red and nuclear staining (DAPI) in blue. The excitation wavelength was 405 nm. Scale bar 20 μm.

Then, RT4 bladder cancer cells with induced expression of CA IX were incubated with the nanoprobe **1**. As shown in Figure 3B, confocal microscopy imaging revealed a specific recognition of membrane CA IX by CAI-Glc-QDs **1** as early as after 1 h of incubation at the concentration of 200 μg/mL, with no relevant signals at lower concentrations (Figure S8B and F, Supporting Information). Then, as proof of the CAI-mediated specific labeling of the membrane, DHLA-EDADA-coated CdSe/ZnS QDs **15**(25) (Figures S9 and S10, Supporting Information) were used as control. No signal was detected, confirming the specificity of our probe (Figures 3C and S8).

In addition, when HT1376 bladder cancer cells, which do not express CA IX on their surface (Figures 3D and S6B, Supporting Information), were incubated with the nanoprobe **1**, no signal was detectable (Figure 3E), thus further supporting the selective and specific CA IX-targeting capacity of the fluorescent probe **1**.

## Conclusions

We designed and prepared a QDs-based fluorescent glyconanoprobe that allowed the selective targeting of the membrane-anchored hCA IX. The nanoprobe was coated with a binary mixture of ligands, compounds **2** and **3**, that do not affect the biology of the target cells, whereas, of note, the CAI-containing ligand **2** maintained the high affinity for the selected tumor-associated biomarker. Confocal microscopy experiments showed that the CAI-Glc-QDs nanoprobe **1** specifically binds the surface of bladder cancer cells according to the expression of the membrane-associated hCA IX, thus suggesting the possibility to exploit such probes for the bioimaging of cancer cells by the selective targeting of these relevant biomarkers.

## Experimental Methods

### Materials and Methods

Reagents were purchased commercially from Sigma-Aldrich and used without any further purification. Varian Cary 4000 UV–vis spectrophotometer (1.0 cm cell) was used to record the UV–vis spectra. A Jasco FP750 spectrofluorimeter (1.0 cm cell) was used to record the fluorescence spectra. Varian Inova 400, Mercury plus 400, and Gemini 200 instruments were used to record the NMR spectra. LC-MS LCQ Fleet ThermoFisher Scientific was used to record the ESI-MS spectra.

### Synthesis of Compound 13

To a stirred solution of **6** (202 mg, 0.399 mmol) and **4** (76 mg, 0.173 mmol) in dry DMF (0.6 mL), CuSO_4_ (11 mg, 0.069 mmol) and sodium ascorbate (17 mg, 0.087 mmol) were added. The mixture was stirred at room temperature for 2 h under a nitrogen atmosphere then the solvent was removed under vacuum by co-evaporation with toluene (3 × 1.5 mL). The crude was purified by flash chromatography on silica gel (dichloromethane/methanol 10:1 and 8:1) to afford product **13**, which was subsequently treated with QuadraSil resin to remove copper traces affording 155 mg of pure **13** (62% yield). ESI-MS (*m*/*z*) calculated for C_60_H_92_N_10_NaO_27_S_2_ [M + Na]^+^ 1471.55, found 1471.01. ^1^H-NMR (400 MHz, CD_3_OD, δ): 7.93 (s, 2 H, H-12), 5.25 (t, *J* = 9.6 Hz, 2 H, H-3′), 5.02 (t, *J* = 9.8 Hz, 2 H, H-4′), 4.88 (dd, *J* = 9.8 Hz*, J* = 8.2 Hz, 2 H, H-2′), 4.73 (d*, J =* 8.0 Hz, 2 H, H-1′), 4.60–4.54 (m, 4 H, H-13), 4.5 (s, 4 H, H-11), 4.30–4.26 (A part of an ABX system, *J* = 4.4 Hz, *J* = 12.4 Hz, 2 H, H-6′a), 4.15–4.12 (B part of an ABX system, *J* = 2.6 Hz, *J* = 12.2 Hz, 2 H, H-6′b), 3.94–3.84 (m, 8 H, H-14, H-18, H-5′), 3.74–3.66 (m, 2 H, H-18), 3.63–3.53 (m, 13 H, H-3, H-15, H-16, H-17), 3.30 (s, 4 H, H-10), 3.25 (t, *J* = 6 Hz, 2 H, H-8), 3.21–3.05 (m, 2 H, H-1), 2.71 (t, *J* = 6.2 Hz, 2 H, H-9), 2.50–2.40 (m, 1 H, H-2), 2.17 (t, *J* = 7.4 Hz, 2 H, H-7), 2.04 (s, 6 H, CH_3_), 2.00 (s, 12 H, 2 CH_3_), 1.96 (s, 6 H, CH_3_), 1.93–1.83 (m, 1 H, H-2), 1.76–1.53 (m, 4 H, H-4, H-6), 1.51–1.37 (m, 2 H, H-5); ^13^C-NMR (100 MHz, CD_3_OD, δ): 174.7, 171.9, 170.9, 170.2, 169.81, 169.75, 144.5, 123.5, 100.5, 72.8, 71.4, 70.2, 70.0, 69.9, 68.99, 68.95, 68.4, 61.7, 58.3, 56.2, 54.7, 50.0, 39.9, 37.9, 37.1, 35.5, 34.3, 34.1, 28.5, 25.2, 19.32, 19.28, 19.16, 19.15.

#### Synthesis of Compound **5**

To an ice-cooled solution of **11** (147 mg, 0.483 mmol) in dry DMF (1.0 mL), *N*-methyl morpholine (79 μL, 0.725 mmol) and CDI (118 mg, 0.725 mmol) were added, and the reaction mixture was stirred for 10 min at 0 °C and for 20 min at room temperature. Then, **12** (194 mg, 0.966 mmol) was added and the reaction mixture stirred for additional 2 h. The solvent was removed by co-evaporation with toluene (3 × 1 mL) and the crude was purified by flash chromatography on silica gel (dichloromethane/methanol 10:1) to afford 176 mg of **5** (75% yield). ESI-MS (*m*/*z*): calculated for C_19_H_30_N_6_NaO_7_S [M + Na]^+^ 509.18, found 509.17. ^1^H-NMR (400 MHz, CDCl_3_, δ): 7.83 (d, *J* = 8.4 Hz, 2 H, H-12/H-13), 7.42 (t, *J* = 5.8 Hz, 1 H, NH), 7.33 (d, *J* = 8.0 Hz, 2 H, H-12, H-13), 6.99 (t, *J* = 5.2 Hz, 1 H, NH), 3.71–3.60 (m, 10 H, H-2, H-3, H-4, H-5, H-6), 3.60–3.52 (m, 4 H, H-7, H-10), 3.45–3.39 (m, 4 H, H-1, H-8), 3.10 (s, 2 H, H-9), 2.89 (t, *J* = 6.8 Hz, 2 H, H-11); ^13^C-NMR (100 MHz, CDCl_3_, δ): 167.9, 167.5, 143.9, 140.8, 129.4, 126.3, 70.5, 70.4, 70.3, 70.2, 69.9, 69.4, 50.6, 42.8, 40.3, 39.4, 35.2.

#### Synthesis of Compound **3**

To a stirred solution of **13** (196 mg, 0.135 mmol) in methanol (1.8 mL), K_2_CO_3_ (19 mg, 0.140 mmol) was added and the reaction mixture stirred at room temperature for 24 h. The crude was purified by filtration on silica gel pad (dichloromethane/methanol 2:1) to afford 86 mg of **3** (57% yield). HRMS-ESI (*m*/*z*): calculated for C_44_H_77_N_10_NaO_19_S_2_ [M + H]^+^ 1113.48024, found 1113.47876 δ = −1.327. ^1^H-NMR (400 MHz, CD_3_OD, δ): 7.95 (s, 2 H, H-12), 4.57 (t, *J* = 5.0 Hz, 4 H, H-13), 4.49 (s, 4 H, H-11), 4.30 (d, *J* = 7.6 Hz, 2 H, H-1′), 4.02–3.94 (m, 2 H, H-18), 3.92–3.82 (m, 6 H, H-14, H-6′a or H-6′b), 3.74–3.50 (m, 19 H, H-3, H-15, H-16, H-17, H-18, H-5′, H-6′a or H-6′b), 3.41–3.21 (m, 10 H, H-9, H-10, H-3′, H-4′), 3.21 - 3.04 (m, 4 H, H-1, H-2′), 2.70 (t, *J* = 6.2 Hz, 2 H, H-8), 2.50–2.39 (m, 1 H, H-2), 2.17 (t, *J* = 7.4 Hz, 2 H, H-7), 1.94–1.82 (m, 1 H, H-2), 1.76–1.52 (m, 2 H, H-4, H-6), 1.50–1.36 (m, 2 H, H-5). ^13^C-NMR (100 MHz, CD_3_OD, δ): 174.72, 172.08, 144.47, 123.59, 103.04, 76.58, 73.66, 70.23, 70.02, 68.97, 68.29, 61.37, 58.30, 56.17, 54.68, 50.02, 46.41, 39.92, 37.93, 37.06, 35.50, 34.30, 34.14, 28.50, 25.23, 7.84.

#### Synthesis of Compound **2**

To a stirred solution of **4** (47 mg, 0.104 mmol) and **5** (152 mg, 0.31 mmol) in dry DMF, CuSO_4_ (6.6 mg, 0.04 mmol) and sodium ascorbate (8 mg, 6.60 mmol) were added. The reaction mixture was stirred at room temperature in the dark for 4.5 h. Then, the solvent was removed under vacuum by co-evaporation with toluene (3 × 1.5 mL). The crude was purified by flash chromatography on silica gel (dichloromethane/methanol 4:1) to afford product **2**, which was subsequently treated with Quadrasil resin to remove copper traces to give 102 mg of pure **2** (69% yield). HRMS-ESI (*m*/*z*): calculated for C_58_H_91_N_16_NaO_17_S_4_ [M + H]^+^ 1411.56254, found 1411.55798 δ = −3.234. ^1^H-NMR (400 MHz, CD_3_OD, δ): 7.94 (s, 2 H, H-12), 7.81 (d, *J* = 8.4 Hz, 4 H, H-24 or H-25), 7.39 (d, *J* = 8.4 Hz, 4 H, H-24 or H-25), 4.55 (t, *J* = 5.0 Hz, 4 H, H-13), 4.47 (s, 4 H, H-11), 3.86 (t, *J* = 5.0 Hz, 4 H, H-14), 3.63–3.55 (m, 17 H, H-3, H-15, H-16, H-17, H-18), 3.52 (t, *J* = 5.4 Hz, 4 H, H-19), 3.45 (t, *J* = 7.0 Hz, 4 H, H-22), 3.39–3.32 (m, 8 H, H-10, H-20), 3.23 (t, *J* = 5.4 Hz, 2 H, H-8), 3.19–3.03 (m, 6 H, H-1, H-21), 2.88 (t, *J* = 7.0 Hz, 4 H, H-23), 2.69 (t, *J* = 6.0 Hz, 2 H, H-9), 2.48–2.38 (m, 1 H, H-2), 2.16 (t, *J* = 7.4 Hz, 2 H, H-7), 1.93–1.79 (m, 1 H, H-2), 1.75–1.51 (m, 4 H, H-4, H-6), 1.49–1.34 (m, 2 H, H-5). ^13^C-NMR (100 MHz, CD_3_OD, δ): 174.7, 172.0, 168.1, 144.5, 143.9, 141.6, 129.1, 125.9, 123.5, 70.1, 70.04, 70.0, 69.9, 68.98, 68.97, 58.3, 56.2, 54.7, 50.0, 42.5, 40.2, 39.9, 39.1, 38.0, 37.1, 35.5, 34.7, 34.3, 34.2, 28.5, 25.2.

#### Synthesis of QDs 1

In a Schlenk tube, 4 mL of a solution of **14** (CHCl_3_) was reduced to 2 mL under reduced pressure to reach a final QD concentration of 3.8 μM (Supporting Information). In a separate Schlenk flask, ligands **2** (23.5 mg, 0.016 mmol) and **3** (37 mg, 0.033 mmol) were dissolved in 1:1 mixture of MeOH/H_2_O (2.6 mL). The solution was degassed by three cycles of *vacuum*/argon, then sodium borohydride was added, and the mixture stirred for 1.5 h under an argon atmosphere. Then, HCl (1M solution in H_2_O) was added to achieve pH 7, and the resulting reaction mixture was added to the stirred solution of **14** using a syringe equipped with a 0.22 μm PTFE filter, and washing the flask with an additional 500 μL of Milli-Q water. The mixture was vigorously stirred for 40 min to allow the ligand exchange; then, the water phase was collected, diluted with 1 mL of methanol, and centrifuged (6000 rpm for 5 min). The supernatant was removed, and the precipitate was washed with MeOH (2 × 2 mL) and centrifuged (6000 rpm for 5 min). Finally, the QDs **1** were dissolved in 2.0 mL of water and freeze-dried. λ_em_ = 596 nm (λ_ex_ = 405 nm).

#### Carbonic Anhydrase Inhibition Assay

An applied photophysics stopped-flow instrument was used for assaying the CA-catalyzed CO_2_ hydration activity by following a previously reported experimental protocol.^33^ As reported earlier, CA isoforms used in the assay were recombinant proteins available in-house.^34−36^

#### In Vitro Assay and Confocal Microscopy Analysis

##### Cell culture

Human bladder cancer RT4 and HT1376 cells were obtained from American TYPE CULTURE COLLECTION (ATCC). The cells were cultured in the selected medium (RT4: McCoy’s 5A; HT1376: DMEM), containing penicillin/streptomycin (100 U and 10 mg/mL, respectively) and including 10% fetal bovine serum (FBS-Gibco), and maintained at 37 °C with 5% CO_2_.^34^ The cells were maintained at a low passage and tested regularly for Mycoplasma negativity. For cell proliferation, the cells were seeded in 48-well plates at 10 000 cells/cm^2^, cultured under hypoxic conditions (1% O_2_, 5% CO_2_, in N_2_) in 1% FBS, and treated with increasing concentrations (1–100 μM) of compounds **2** and **3**. After 72 h of incubation, the cells were trypsinized and cell counting was performed with a MACSQuant analyzer (Miltenyi Biotec).

## Supporting Information Available

Synthesis of compounds **4**, **6**, **11**, and of QDs **14**; ^1^H-NMR and ^13^C-NMR spectra of compounds **2**–**6** (Figures S12–S30); TEM and HRTEM micrographs of QDs **1** and **15** (Figures S1, S2, and S9B); Absorbance and emission spectra of QDs **14** (Figure S11); Thermogravimetric analysis; Immunohistochemistry; Absorbance and fluorescence spectra of QDs **15**; Methods for QD concentration; HRMS-ESI of compounds **2** and **3** (Figures S31 and S32) (PDF)

## Abbreviations Used

QDs: quantum dots
hCA: human carbonic anhydrase
CAI: carbonic anhydrase inhibitor
Glc: glucose
ATCC: American type culture collection
DHLA: dihydrolipoic acid
EDADA: ethylenediamine-*N*,*N*-diacetic acid residue
