# Supplementary material for: Glyco-Coated CdSe/ZnS Quantum Dots as Nanoprobes for Carbonic Anhydrase IX Imaging in Cancer Cells
Source: ACS Appl Nano Mater. 2021 Nov 17;4(12):14153–60. doi: 10.1021/acsanm.1c03603 (PMC8713163; doi:10.1021/acsanm.1c03603)
Supplement: Supplementary file 1 — an1c03603_si_001.pdf [file an1c03603_si_001.pdf]

Supporting Information

Glyco-Coated CdSe/ZnS Quantum Dots as Nanoprobes for Carbonic Anhydrase IX Imaging in Cancer Cells

Giacomo Biagiotti,[a,b,‡] *Andrea Angeli,*[c,‡] *Arianna Giacomini,*[d] *Gianluca Toniolo,*[a,b] *Luca Landini,*[a] *Gianluca Salerno,*[a] *Lorenzo Di Cesare Mannelli,*[e] *Carla Ghelardini,*[e] *Tommaso Mello,*[f] *Silvia Mussi,*[d] *Cosetta Ravelli,*[d] *Marcello Marelli,*[g] *Stefano Cicchi,*[a,b] *Enzo Menna,*[h,i,b] *Roberto Ronca,*[d] *Claudiu T. Supuran,*[c,*] *Barbara Richichi.* [a,b,*]

[a] Department of Chemistry 'Ugo Schiff', University of Firenze, Via della Lastruccia 13, 50019 Sesto Fiorentino (FI, Italy).

[b] Consorzio Interuniversitario Nazionale per la Scienza e Tecnologia dei Materiali (INSTM, Via G. Giusti, 9, 50121 Firenze (Italy).

[c] Department of Neuroscience, Psychology, Drug Research and Child Health – NEUROFARBA, Section of Pharmaecutical Chemistry, University of Firenze, Via Ugo Schiff 7, 50019 Sesto Fiorentino, (FI, Italy).

[d] Department of Molecular and translational Medicine, University of Brescia, Viale Europa 11, 25123 Brescia (Italy).

[e] Department of Neuroscience, Psychology, Drug Research and Child Health - NEUROFARBA - Pharmacology and Toxicology Section, University of Firenze, V.le Pieraccini 6, 50139, Firenze (Italy).

[f] Department of Clinical and Experimental Biomedical Sciences "Mario Serio" – Gastroenterology Unit, University of Firenze, V.le Pieraccini 6, 50139 Firenze (Italy).

[g] Istituto di scienze e tecnologie chimiche "Giulio Natta", CNR-SCITEC, Sede Fantoli, Via Fantoli 16/15, 20138 Milano (Italy).

[h] Department of Chemical Sciences, University of Padova, Via Marzolo 1, 35131 Padova (Italy).

[i] Centre for Mechanics of Biological Materials – CMBM, Via Marzolo 9, 35131 Padova (Italy).

Corresponding Author

* Barbara Richichi barbara.richichi@unifi.it

* Claudiu T. Supuran claudiu.supuran@unifi.it

#GB and AA equally contributed to the work

Table of content

**Example of numeration**	S4
**Synthesis of 6**	S4
**Scheme S1**	S4
**Synthesis of QDs 14**	S5
**Scheme S2**	S5
**Figure S1**	S6
**Figure S2**	S6
**Figure S3**	S7
**Figure S4**	S8
**Thermogravimetric analysis (TGA)**	S9
**Figure S5**	S9
**Table S1**	S9
**Immunocytochemistry and incubation with QDs.**	S9
**Figure S6**	S10
**Figure S7**	S10
**Figure S8**	S11
**Figure S9**	S11
**Figure S10**	S12
**Synthesis of 4**	S12
**Synthesis of 11**	S12
**Determination of QDs concentration**	S13
**Figure S11**	S13
**Figure S12**	S14
**Figure S13**	S15
**Figure S14**	S16
**Figure S15**	S17

Figure S16 S18
Figure S17 S19
Figure S18 S20
Figure S19 S21
Figure S20 S22
Figure S21 S23
Figure S22 S24
Figure S23 S25
Figure S24 S26
Figure S25 S27
Figure S26 S28
Figure S27 S29
Figure S28 S30
Figure S29 S31
Figure S30 S32
Figure S31 S33
Figure S32 S34
References S34

Example of numeration

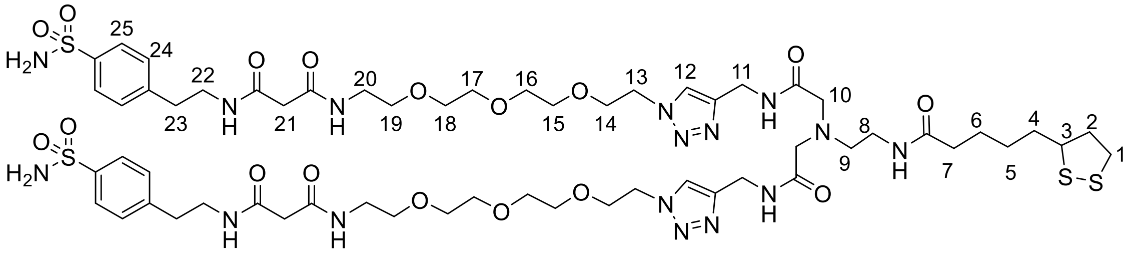

2

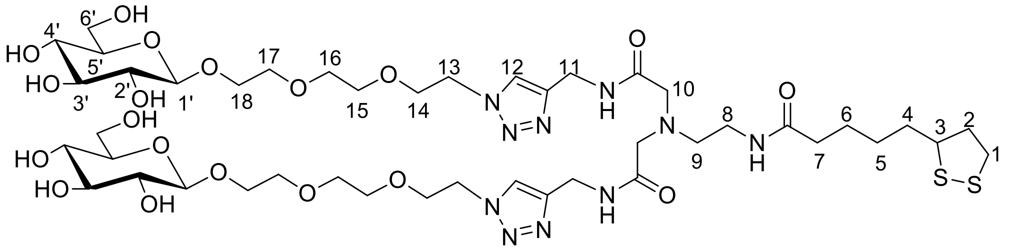

3

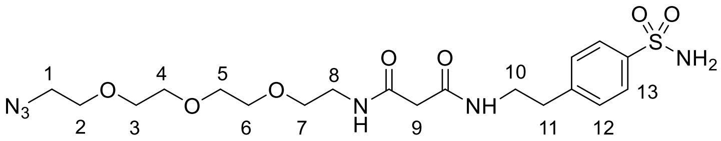

5

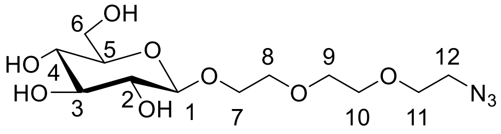

6

Synthesis of 6

Scheme S1. Synthesis of compound **6**.

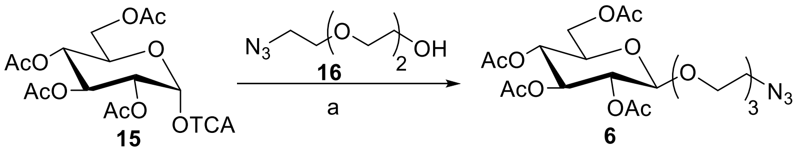

To an ice-cooled solution of **15**[1] (797 mg, 1.61 mmol) and **16**[2] (340 mg, 1.94 mmol) in dry dichloromethane, trimethylsilyl trifluoromethanesulfonate (53 mg, 0.240 mmol) was added. The reaction mixture was stirred for 10 minutes at 0°C, then it was warmed to room temperature and stirred for 50 minutes. Then, triethylamine (100 µL, 0.72 mmol) was added to reach pH = 7. The

mixture was diluted with DCM (3 mL) and acetic anhydride (157 µL, 0.322 mmol), pyridine (130 µL, 1.61 mmol) and 4-dimethylaminopyridine (39 mg, 0.322 mmol) were added. The reaction mixture was stirred for 1 h at room temperature, then diluted with dichloromethane (180 mL) and washed with a saturated solution of NH_4Cl (2 x 20 mL) and Brine (1 x 20 mL). The organic phase was dried on Na_2SO_4, filtered and the solvent was removed under *vacuum*. The crude was purified by flash chromatography on silica gel (ethyl acetate/petroleum ethers 2:1) affording 562 mg of **6** as β-anomer (69% yield). ^{1}H NMR (400 MHz, $CDCl_3$, δ): 5.18 (at, *J* = 9.6, 1 H, H-3), 5.06 (at, *J* = 9.6 Hz, 1 H, H-4), 4.96 (dd, *J*= 9.6 Hz, *J* = 8.2 Hz, 1 H, H-2), 4.59 (d, *J*= 8.0 Hz, 1 H, H-1 β), 4.25-4.21 (A part of ABX system, *J* = 4.4 Hz, *J*= 12.4 Hz, 1 H, H-6a), 4.14-4.07 (B part of ABX system, *J* = 2.0 Hz, *J* = 12.4 Hz, 1 H, H-6b), 3.96 - 3.88 (m, 1 H, H-7), 3.80 - 3.54 (m, 10 H, H-5, H-7, H-8, H-9, H-10, H-11), 3.38 (t, J_1= 4.8 Hz, 2 H, H-12), 2.06 (s, 3 H, CH_3), 2.02 (s, 3 H, CH_3), 2.00 (s, 3 H, CH_3), 1.98 (s, 3 H, CH_3).^{13}C-NMR (100 MHz, $CDCl_3$, δ): 170.6, 170.2, 169.4, 169.3, 100.8 (C1), 72.8 (C3), 71.7 (C5), 71.3 (C2), 70.67, 70.65, 70.4, 70.0 (C8, C9, C10, C11), 69.0 (C7), 68.4 (C4), 61.9 (C6), 50.6 (C12), 20.7, 20.6, 20.58, 20.56 ppm (4 CH_3). NMR data are in agreement with the literature.[3]

Synthesis of QDs 14

Scheme S2. Synthesis of TOPO-coated core shell CdSe/ZnS QDs **14**. Reaction conditions: a) Trioctyl phosphine oxide (TOPO), tributyl phosphine (TBP), Se, 300°C under argon atmosphere in the dark; b) Zinc stearate, hexamethyldisilathiane, TOPO, Toluene, 180°C under argon atmosphere in the dark.[4–7]

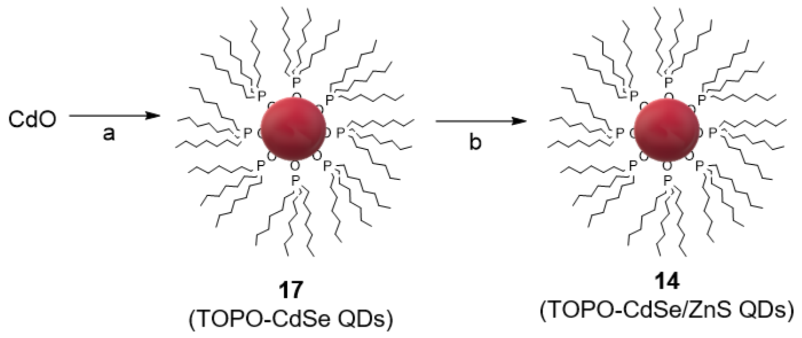

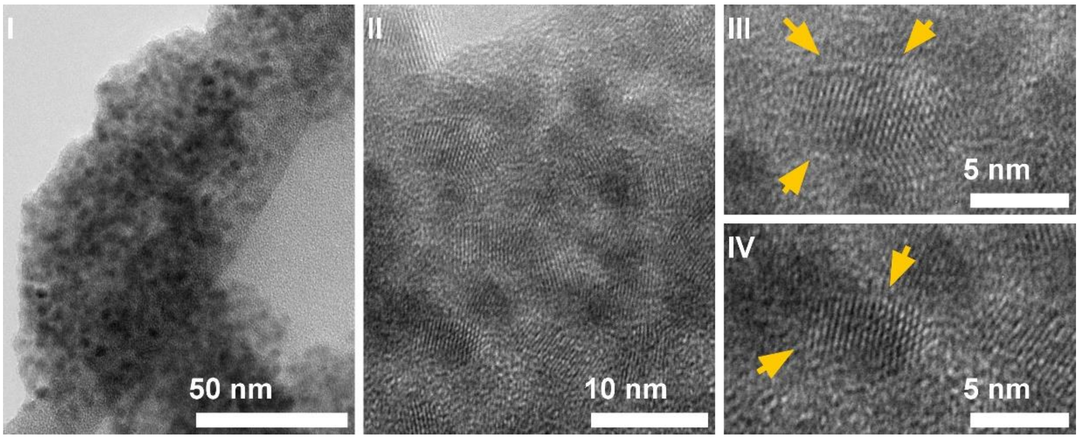

Figure S1. Representative (**I**) TEM and (**II**) HRTEM micrographs of CAI-Glc-QDs **1** and related magnifications (**III** and **IV**) of elongated QDs, arrows highlight the edges and the shape.

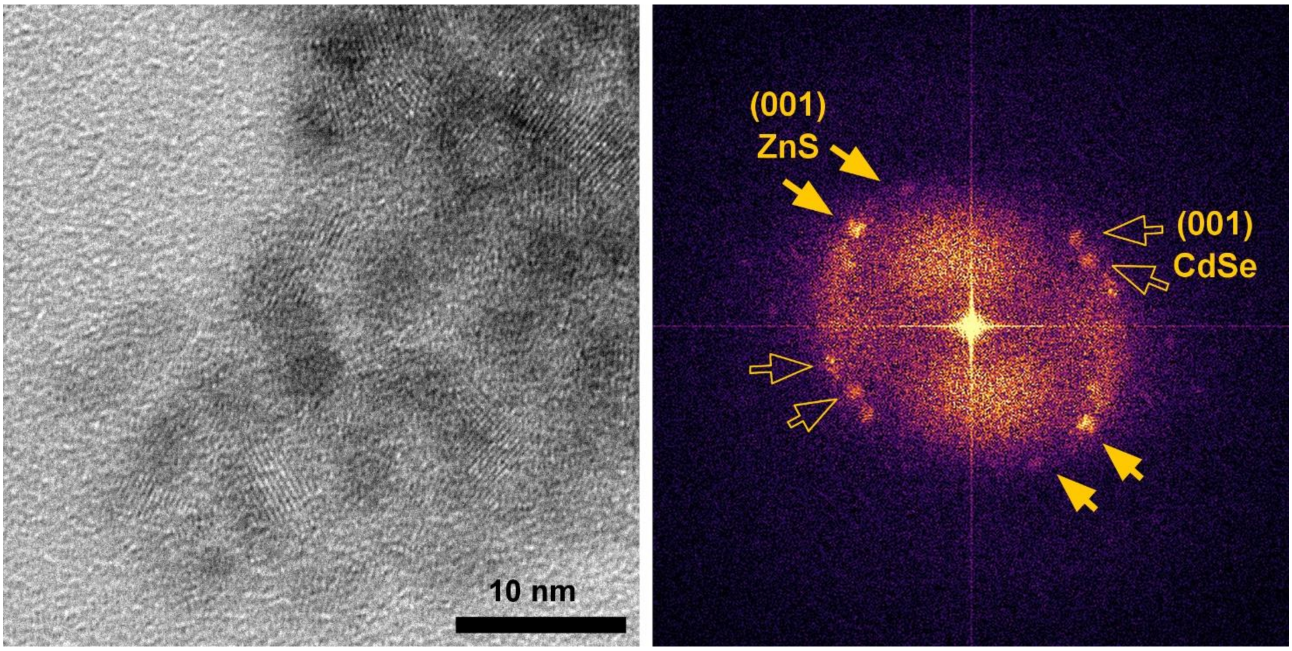

Figure S2. Representative HRTEM micrograph of CAI-Glc-QDs **1** and related FFT analysis (false-color image): outlined arrows point to CdSe (001) indexed reflexes and solid arrows point to ZnS (001) indexed reflexes.

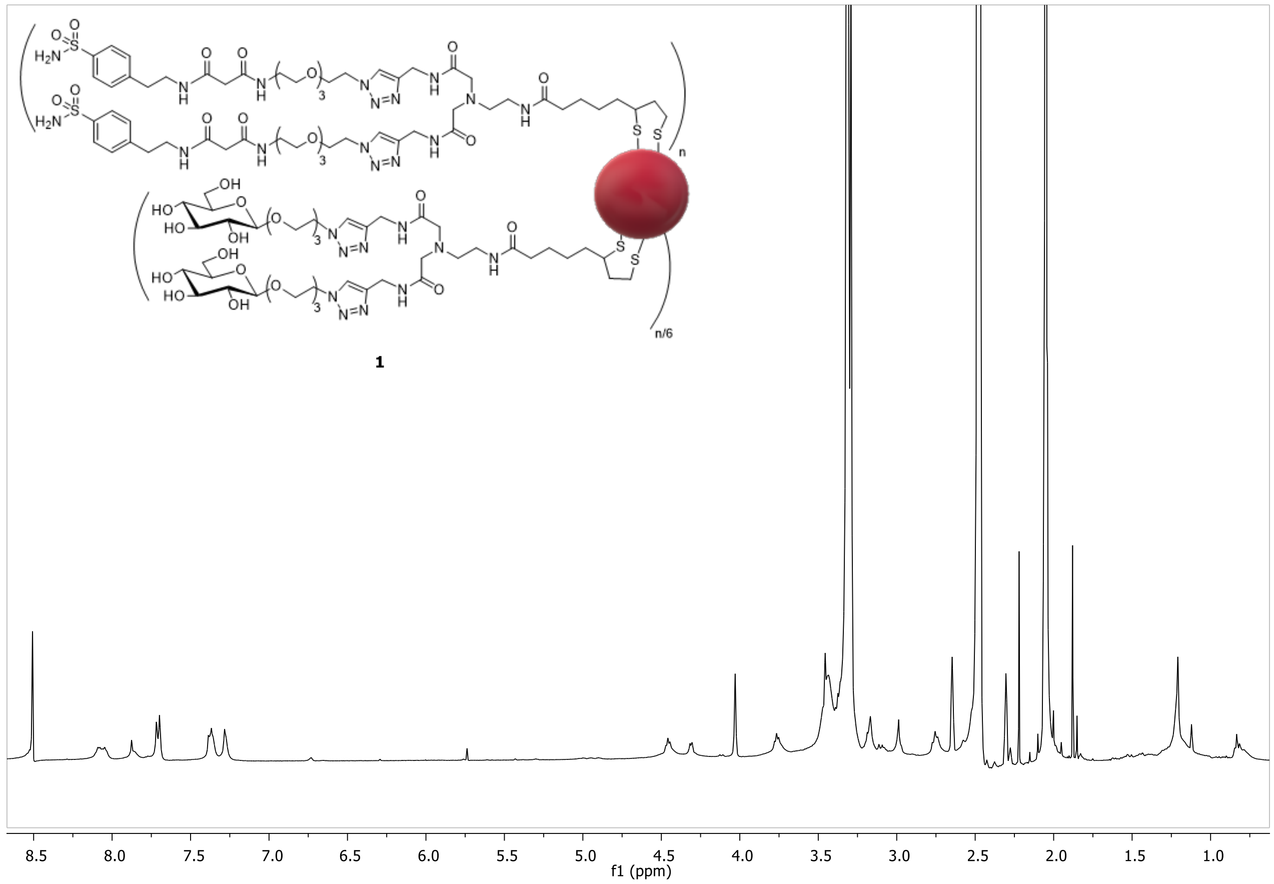

Figure S3. ^{1}H-NMR (400 MHz DMSO-d6) of QDs **1.**

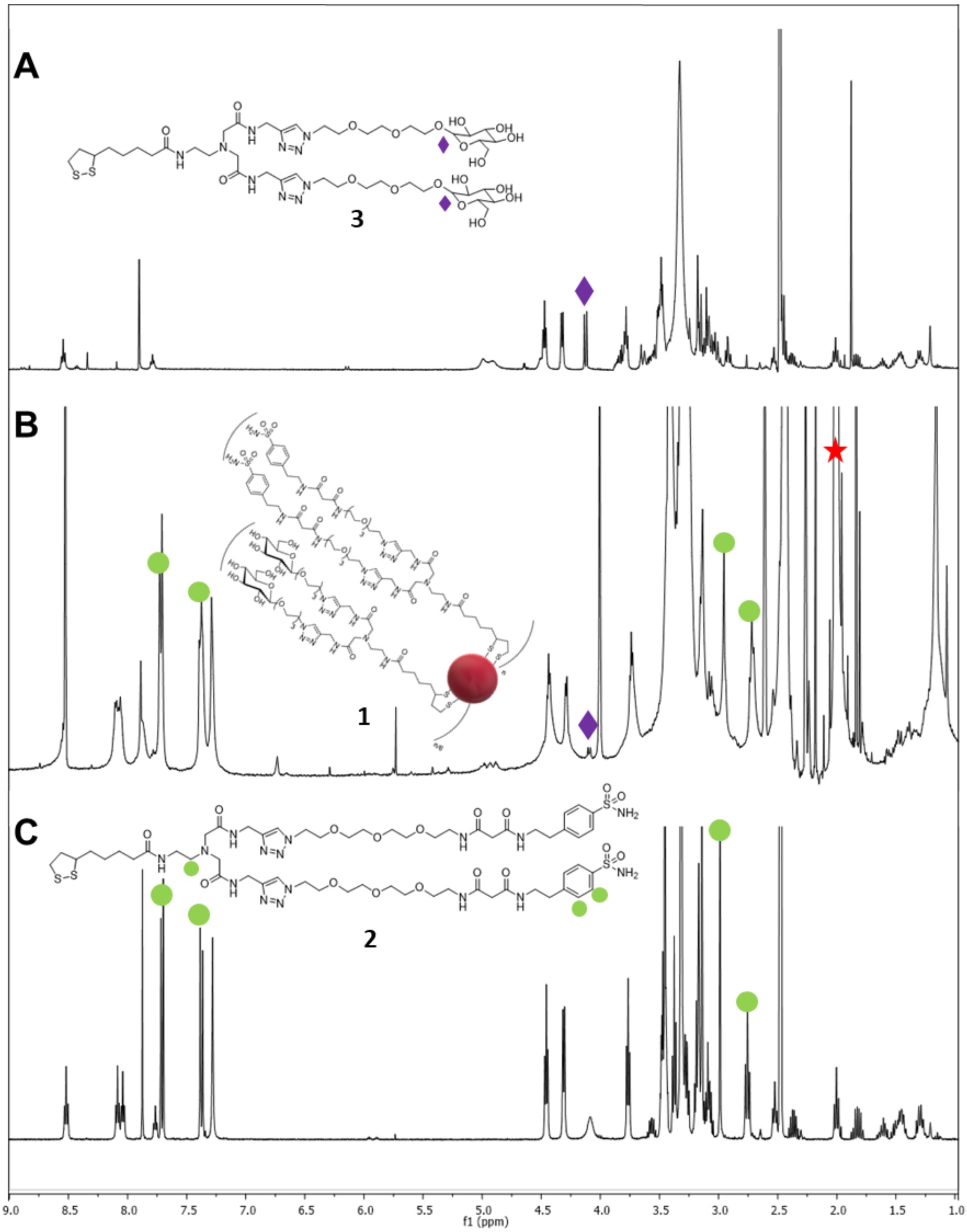

Figure S4. ^{1}H-NMR (DMSO-d6, 400 MHz) of: A) Compound **3**; B) QDs **1**; C) Compound **2.** Purple diamonds indicate the signal of anomeric H-1 of Glc, green dots indicate the signals of protons related to the sulfonamide and red star indicates the signals of acetonitrile (used as internal standard).

Thermogravimetric analysis (TGA)

The thermal properties were characterized by thermogravimetric analysis performed in a TGA Q5000IR, programmed in a heating rate of 10°C/minute from 50 to 900°C with a nitrogen flow of 25 mL/minute.

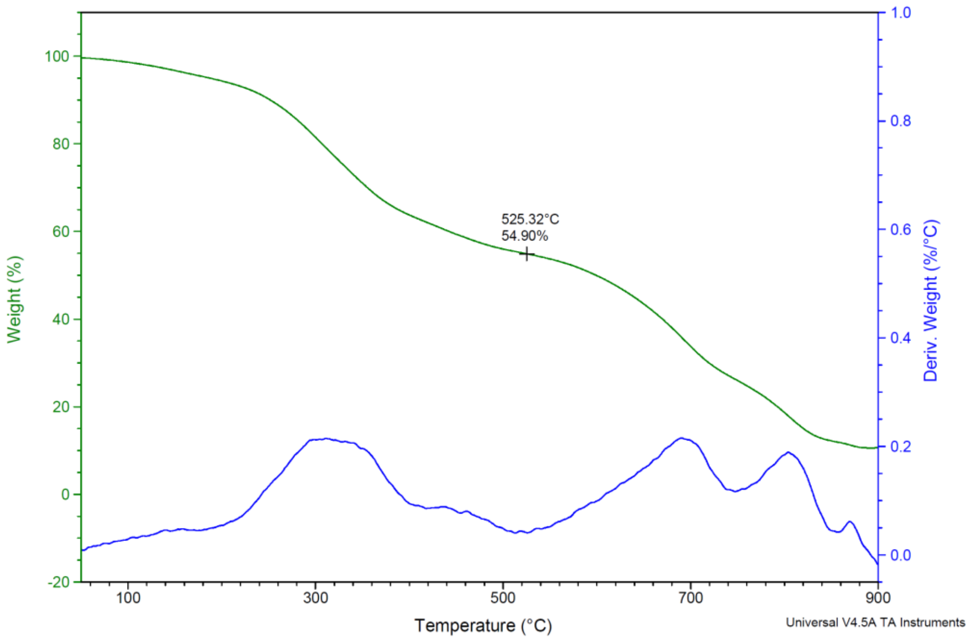

Figure S5. Thermogravimetric analysis of CAI-Glc-QDs **1**.

Table S1. The percentages of ligand weight on QDs characterized by NMR and TGA.

QDs	NMR	TGA
**1**	40.9 (**2**), 5.6 (**3**)	45% (**2**+**3**)

Immunocytochemistry and incubation with QDs.

For immunocytochemistry, RT4 and HT1376 cells were seeded in µ-Slide 8well ibiTreat chambers (Ibidi), 2 x 10^5 cells/well and incubated for 24 hours under hypoxic conditions (1% O_2, 5% CO_2, in N2). Cells were stained for CAIX with the M75 anti-CAIX antibody (kindly provided by Dr. S. Pastorekova), using a chicken anti-mouse Alexafluor594 secondary antibody (Invitrogen).

For imaging with QDs preparations RT4 and HT1376 cells were seeded in µ-Slide 8well ibiTreat chambers (Ibidi), 2 x 10^5 cells/well and incubated for 24 hours under hypoxic conditions. Cells were incubated for 1 hour with 200 µg/mL QDs (**1** and **15**), for staining of the nuclei DAPI was added in the last 20 minutes of incubation, then all wells were extensively washed with PBS and imaged immediately thereafter. Imaging was performed on a Zeiss LSM510 Meta confocal microscope equipped with a Plan-Apochromat 63x/1.4 NA oil objective. The excitation source was a 405 diode

laser for both DAPI and QDs which were acquired at 405-515 nm and 550-625 nm respectively. Z-stack sectioning and maximum intensity projections (MIP) were obtained using Zen Black 2 software (Zeiss).

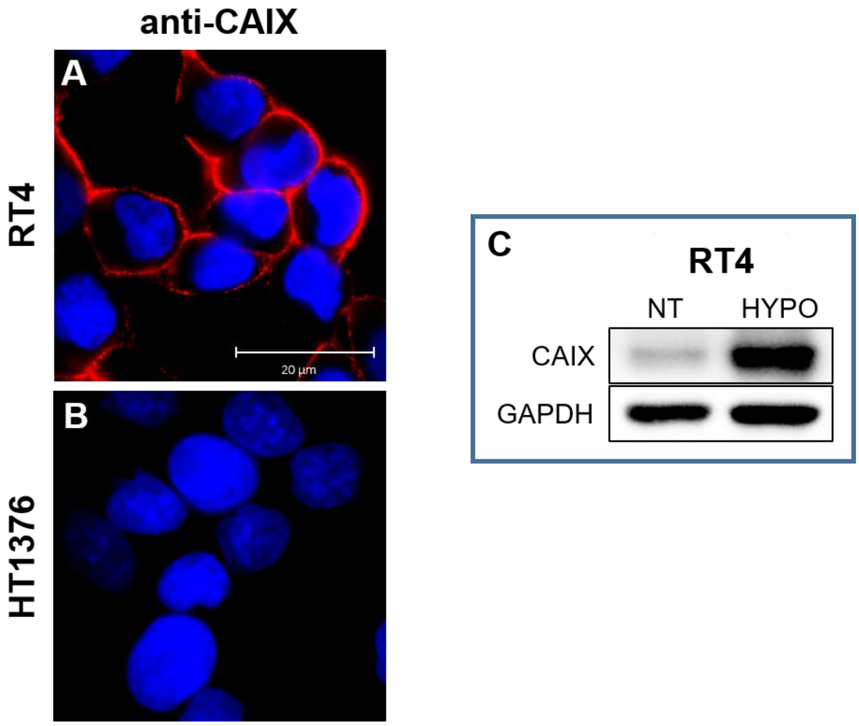

Figure S6. Immunocytochemistry on RT4 (**A**) and HT1376 (**B**) bladder cancer cells. CA IX specific staining in red and nuclear staining (DAPI) in blue. Scale bar 20μm. Western blot (**C**) for CA IX expression in RT4 cells under normoxic (NT) and hypoxic (HYPO) culture conditions.

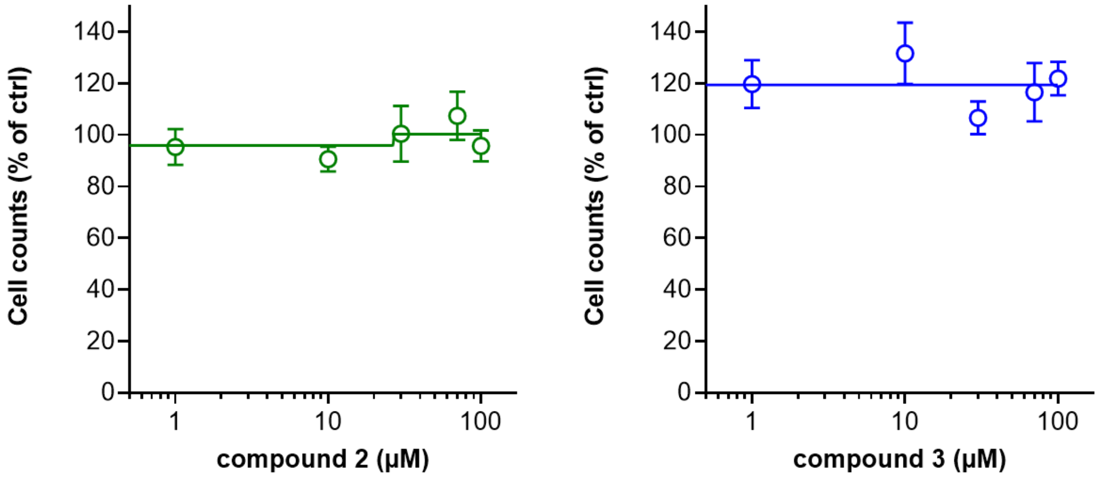

Figure S7. *In vitro* cell proliferation assay performed on RT4 bladder cancer cells incubated with compound **2** and compound **3** at different concentrations for 72 hours.

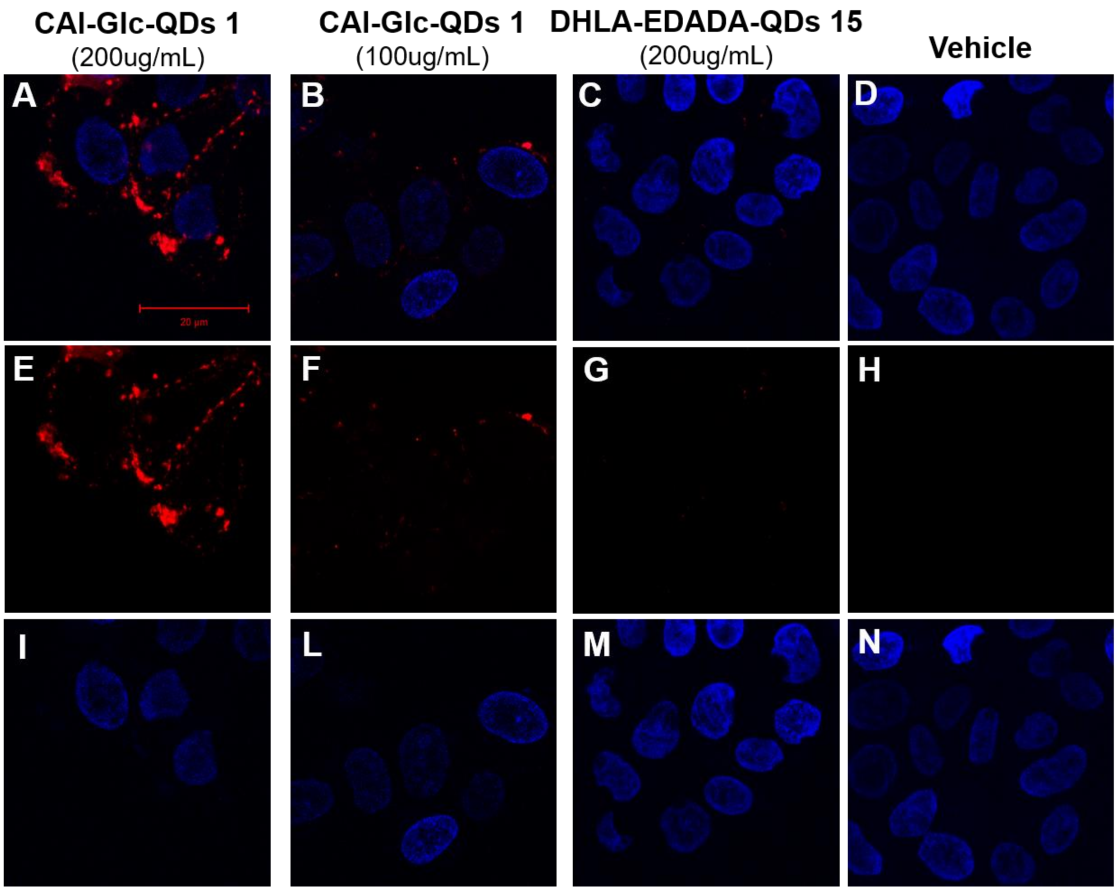

Figure S8. *In vitro* confocal microscopy imaging of RT4 bladder cancer cells incubated with CAI-Glc-QDs **1** at 200µg/ml (**A**, **E** and **I**), CAI-Glc-QDs **1** at 100µg/ml (**B**, **F** and **L**), DHLA-EDADA-QDs **15** at 200µg/ml (**C**, **G** and **M**), and vehicle (**D**, **H** and **N**). QDs fluorescence in red and nuclear staining (DAPI) in blue. Excitation wavelength of 405 nm. Scale bar 20µm.

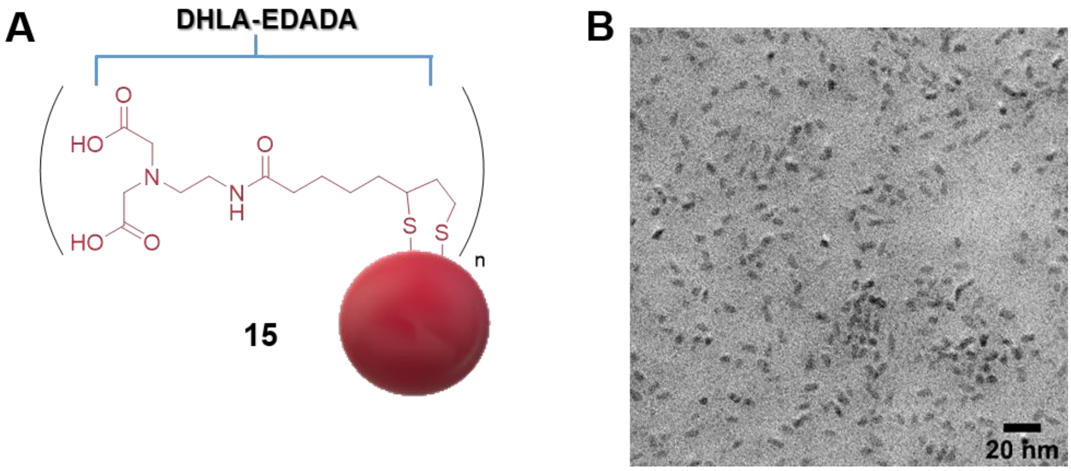

Figure S9. **A**. Schematic representation of DHLA-EDADA coated CdSe/ZnS QDs **15.**[8] **B**. Representative TEM migrograph of DHLA-EDADA coated CdSe/ZnS QDs **15**.

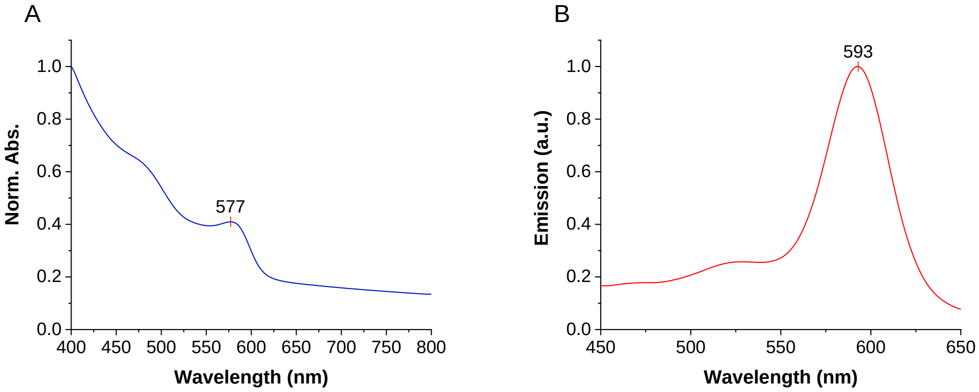

Figure S10. A. Absorbance spectrum of DHLA-EDADA coated CdSe/ZnS QDs **15** in H_2O; B. Emission spectrum (λ_{exc} = 405 nm) of DHLA-EDADA coated CdSe/ZnS QDs **15** in H_2O.

Synthesis of 4

To an ice-cooled solution of **7** (262 mg, 0.720 mmol) in dry DMF, TBTU (832 mg, 2.60 mmol) and *N*-methyl-morpholine (286 µL, 2.60 mmol) were added. The reaction mixture was warmed at room temperature and stirred for 20 minutes. Then, the reaction mixture was cooled to 0°C, and propargylamine (276 µL, 4.31 mmol) was added. The reaction mixture stirred at room temperature for 24 h. The solvent was removed by co-evaporation with toluene (3 x 2 mL) under *vacuum*, then the solid was dissolved in DCM (200 mL) and washed with water (3 x 20 mL) and Brine (1 x 20 mL). The organic phase was dried on Na_2SO_4, filtered and the solvent was removed under *vacuum*. The crude was purified by flash chromatography on silica gel (dichloromethane/methanol 18:1) affording 238 mg of **4** (75% yield). ESI-MS (*m/z*): calculated for $C_{20}H_{30}N_4NaO_3S_2$ $[M+Na]^+$ 461.17, found 461.25. ^{1}H NMR (400 MHz, $CDCl_3$, δ): 7.60 (bs, 2 H, NH), 6.85 (bs, 1 H, NH), 4.05 (dd, *J* = 5.4 Hz, *J* = 2.7 Hz, 4 H, H-11), 3.62 - 3.52 (m, 1 H, H-3), 3.38 – 3.24 (m, 6 H, H-8, H-10), 3.22 - 3.06 (m, 2 H, H-1), 2.73 (m, 2 H, H-9), 2.51 - 2.41 (m, 1 H, H-2), 2.32 - 2.21 (m, 4 H, H-7, H-12), 1.96 - 1.84 (m, 1 H, H-2), 1.79 - 1.59 (m, 4 H, H-4, H-6), 1.56 - 1.39 (m, 2 H, H-5). ^{13}C-NMR (100 MHz, $CDCl_3$, δ): 173.8, 170.7, 79.5, 71.6, 59.1, 56.5, 56.3, 40.3, 38.4, 37.7, 36.4, 34.6, 28.97, 28.96, 25.4.

Synthesis of 11

To a solution of **9** (495 mg, 2.27 mmol) in dry dichloromethane (1.9 mL), Meldrum acid **10** (138 mg, 0.960 mmol) was added and then the reaction mixture was warmed at 40°C and stirred for 18 h. Then, the reaction mixture was diluted with dichloromethane (180 mL) and washed with a 1 M solution of HCl (3 x 10 mL). The aqueous phases were collected and extracted with dichloromethane (5 x 70 mL), then the organic phases were collected, dried on Na_2SO_4, filtered and the solvent was removed under *vacuum* to obtain 281 mg of **11** (97% yield). The product was used without further purification for the next synthetic step. ESI-MS (*m/z*) calculated for $C_{11}H_{19}N_4O_6^-$ $[M-H]^-$ 303.13, found 302.99.

^{1}H NMR (400 MHz, $CDCl_3$, δ): 3.71 - 3.64 (m, 10 H, H-2, H-3, H-4, H-5, H-6), 3.62 - 3.58 (m, 2 H, H-7), 3.52 (m, 2 H, H-8), 3.42 (t, $J = 5$ Hz, 2 H, H-1), 3.33 (s, 2 H, H-9). ^{13}C-NMR (100 MHz, $CDCl_3$, δ): 169.8, 168.0, 70.32, 70.29, 70.2, 69.98, 69.7, 69.0, 50.4, 39.5, 39.4.

Determination of QDs concentration

QDs concentration was estimated according to the literature,[9] the diameter of nanoparticles was evaluated either by TEM imaging and maximum emission wavelength.[10]

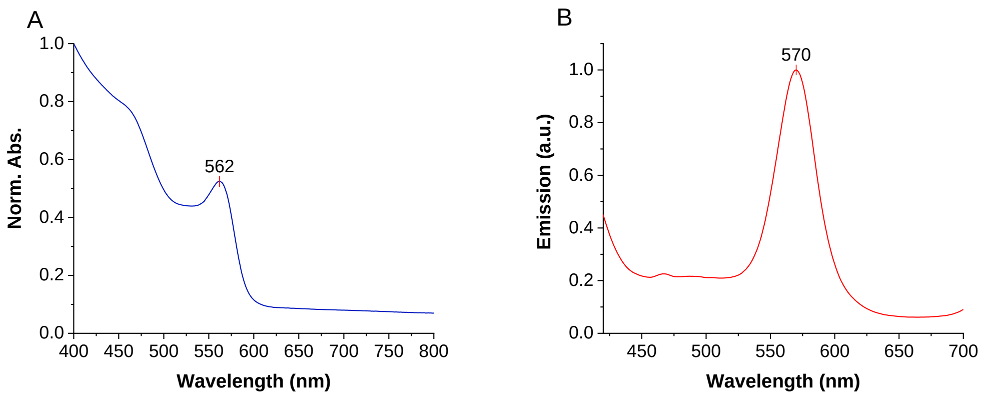

Figure S11. A) Absorbance spectrum of TOPO-CdSe/ZnS QDs **14** in chloroform; B) Emission spectrum (λ_{exc} = 405 nm) of TOPO-CdSe/ZnS QDs **14** in chloroform.

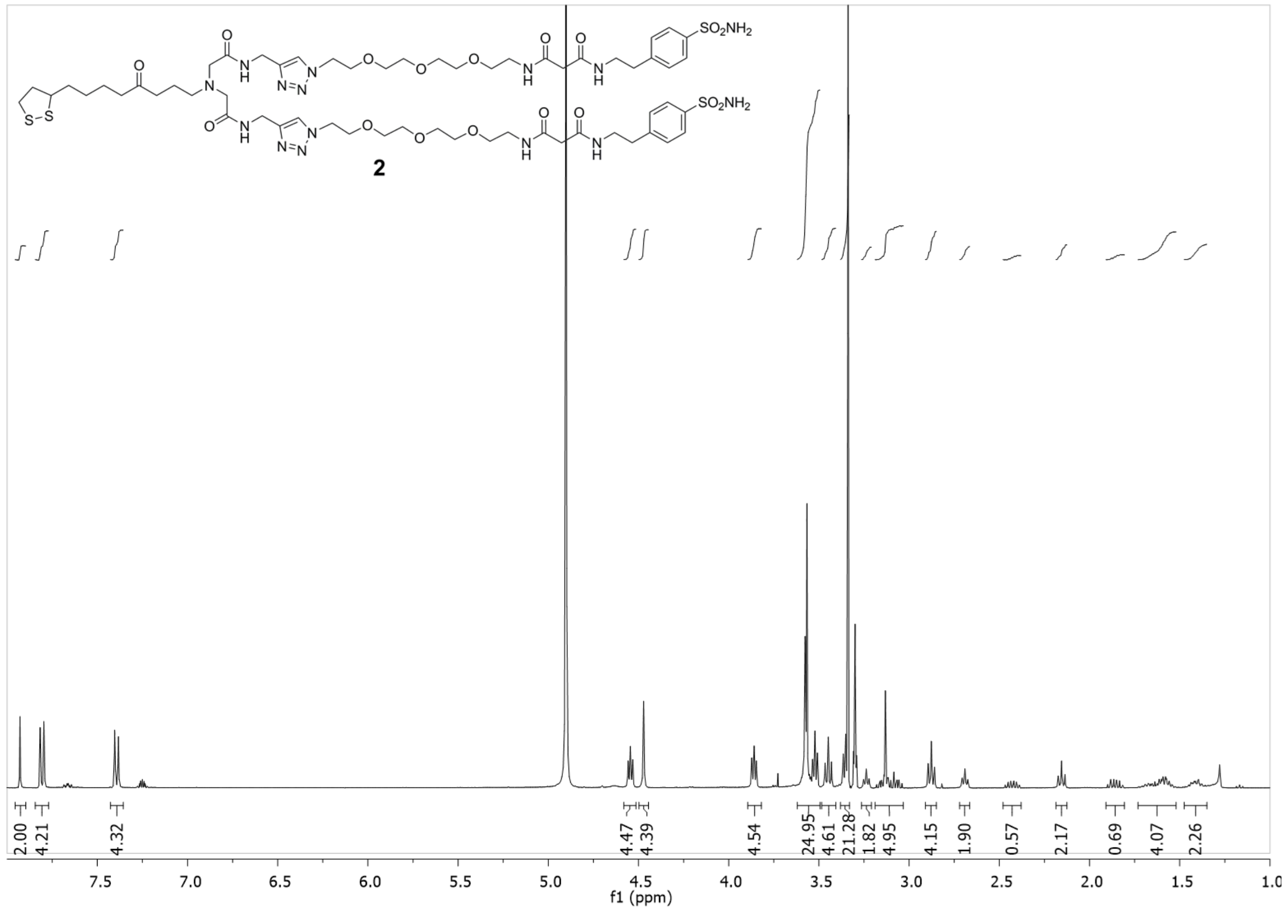

Figure S12. ^{1}H-NMR (400 MHz, CD_3OD) of **2.**

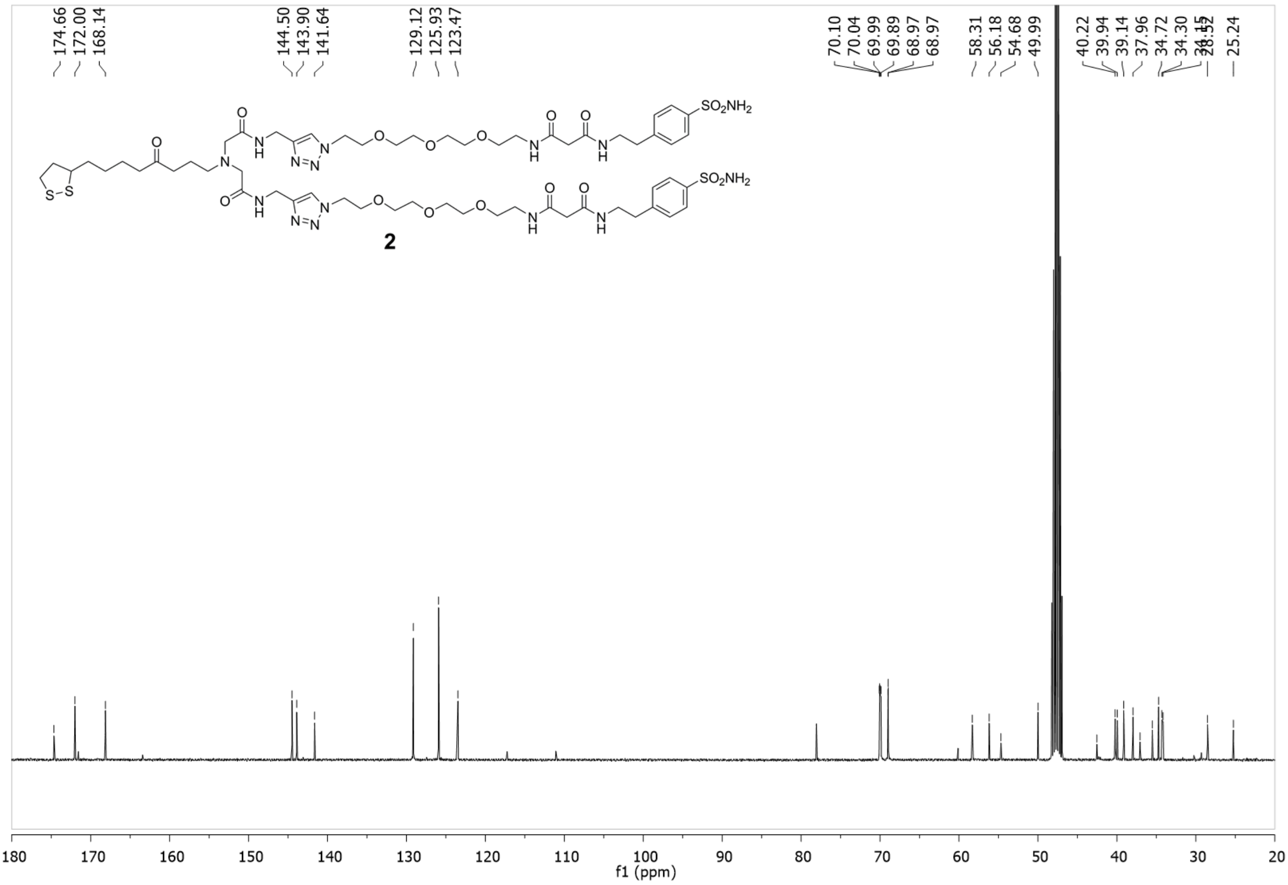

Figure S13. ^{13}C-NMR (100 MHz, CD_3OD) of **2.**

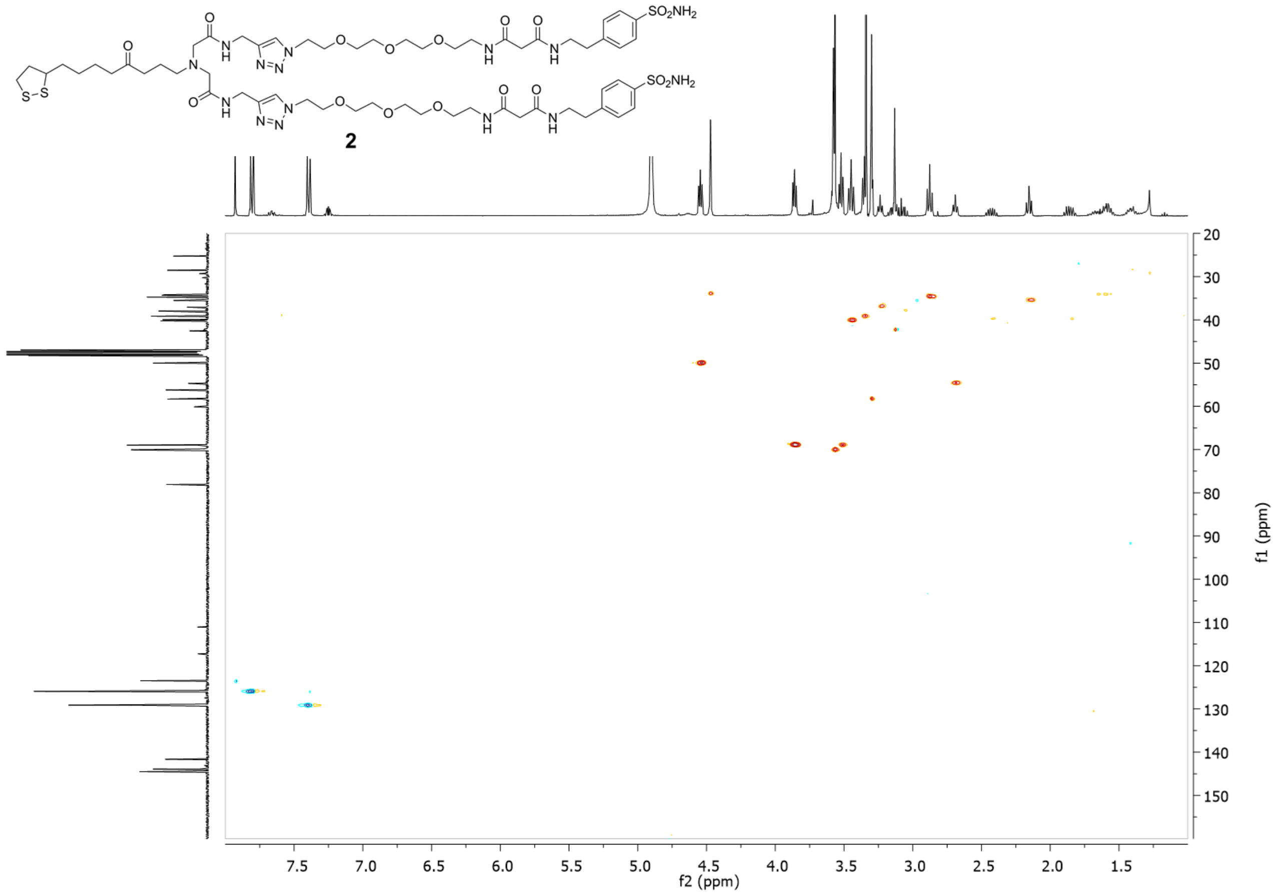

Figure S14. gCOSY-NMR (400 MHz, CD_3OD) of **2.**

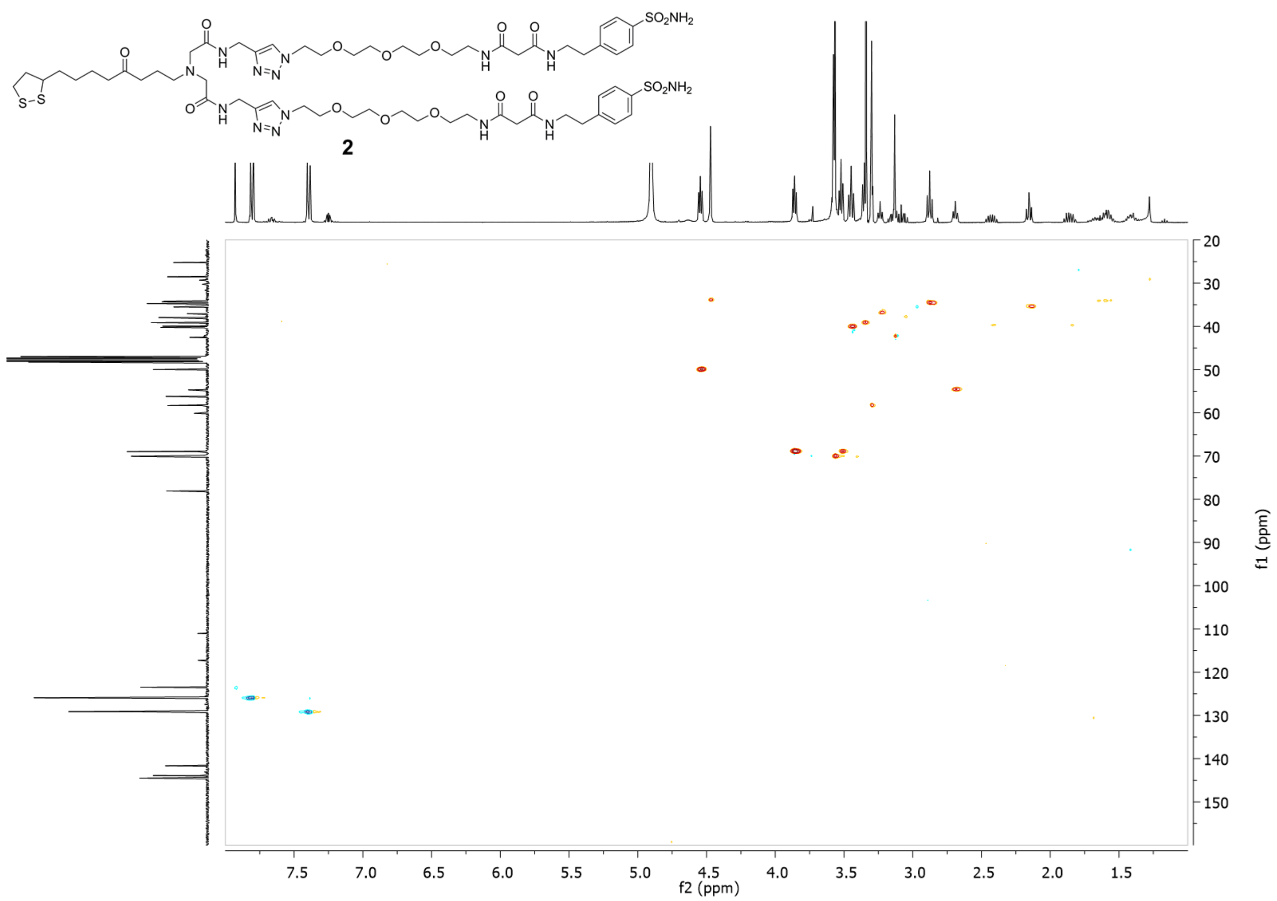

Figure S15. gHSQC-NMR (400 MHz, CD_3OD) of **2.**

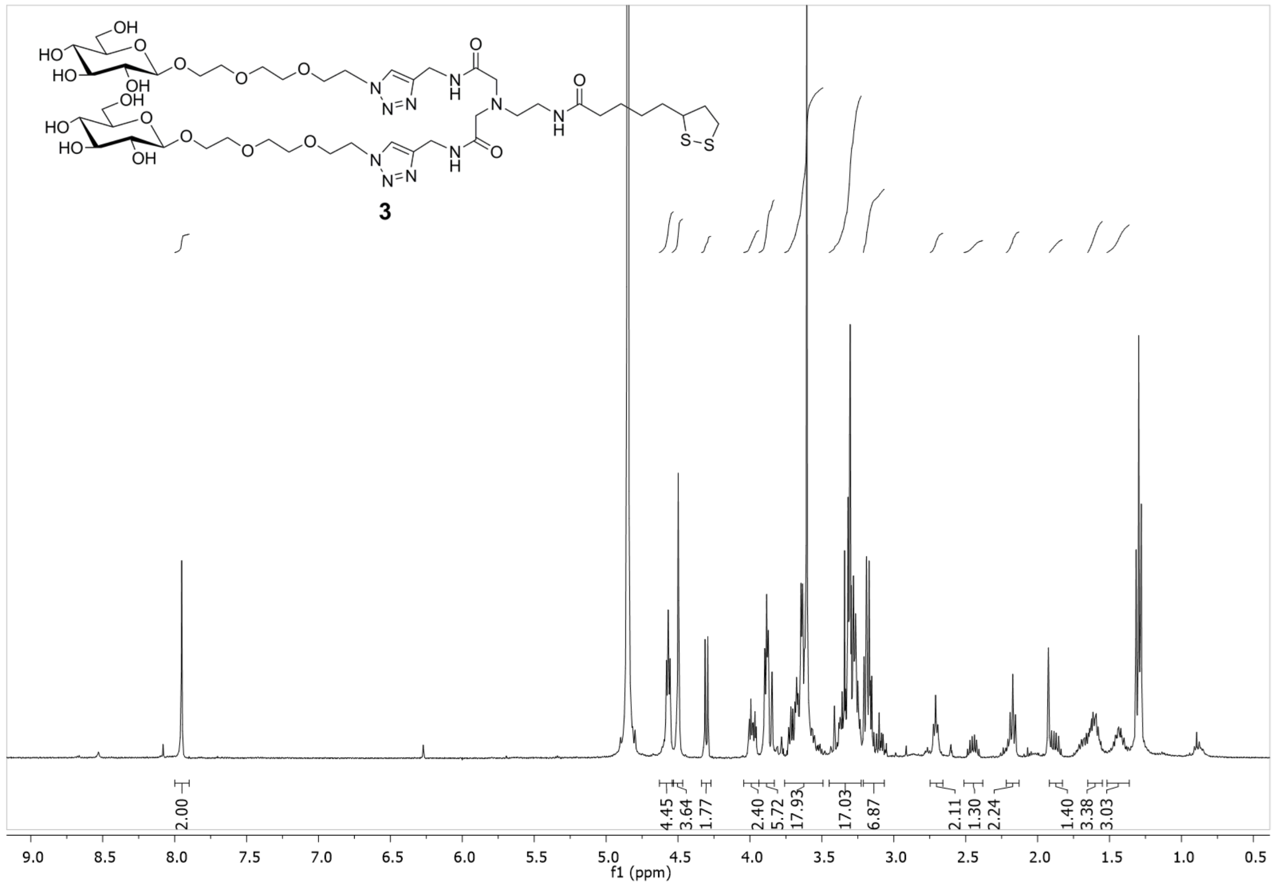

Figure S16. ^{1}H-NMR (400 MHz, CD_3OD) of **3.**

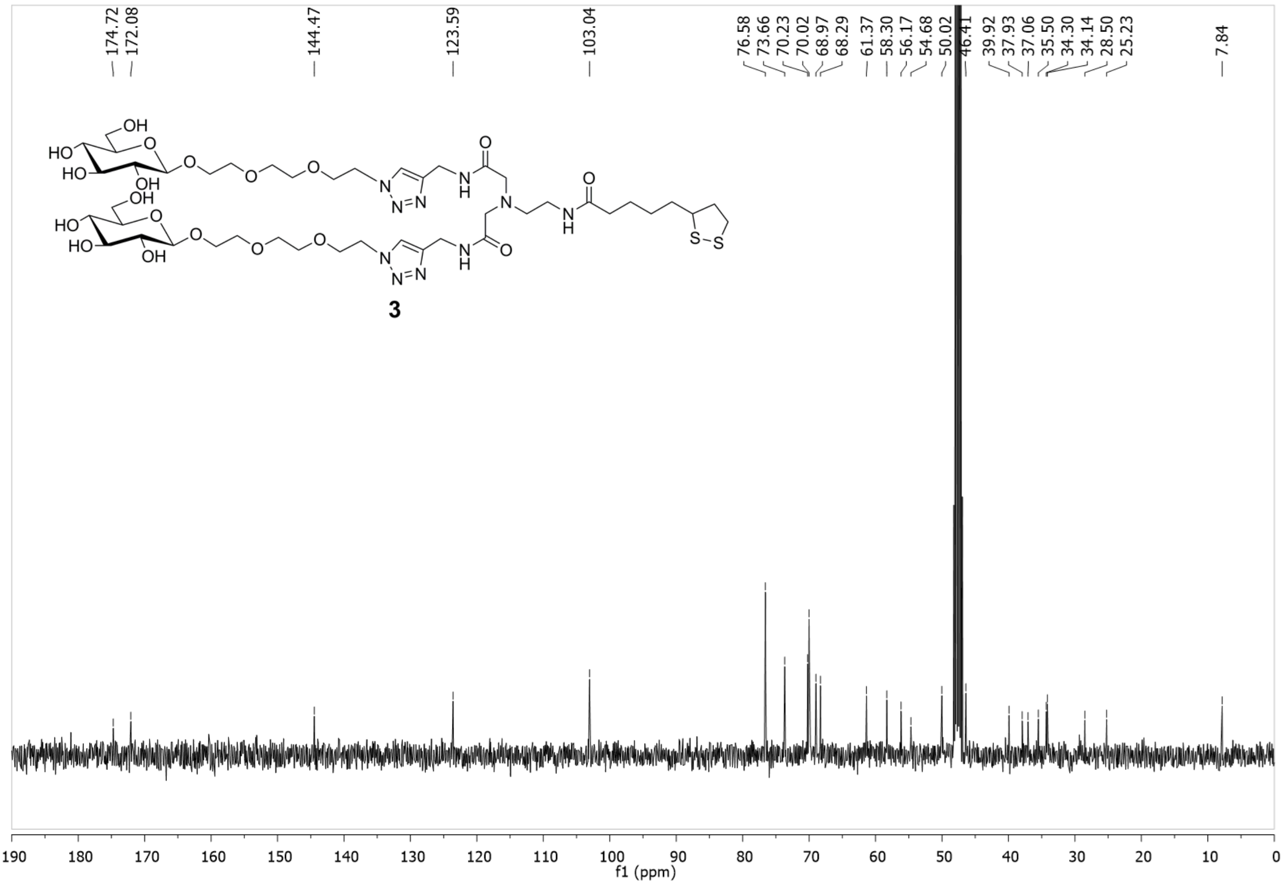

Figure S17. ^{13}C-NMR (100 MHz, CD_3OD) of **3.**

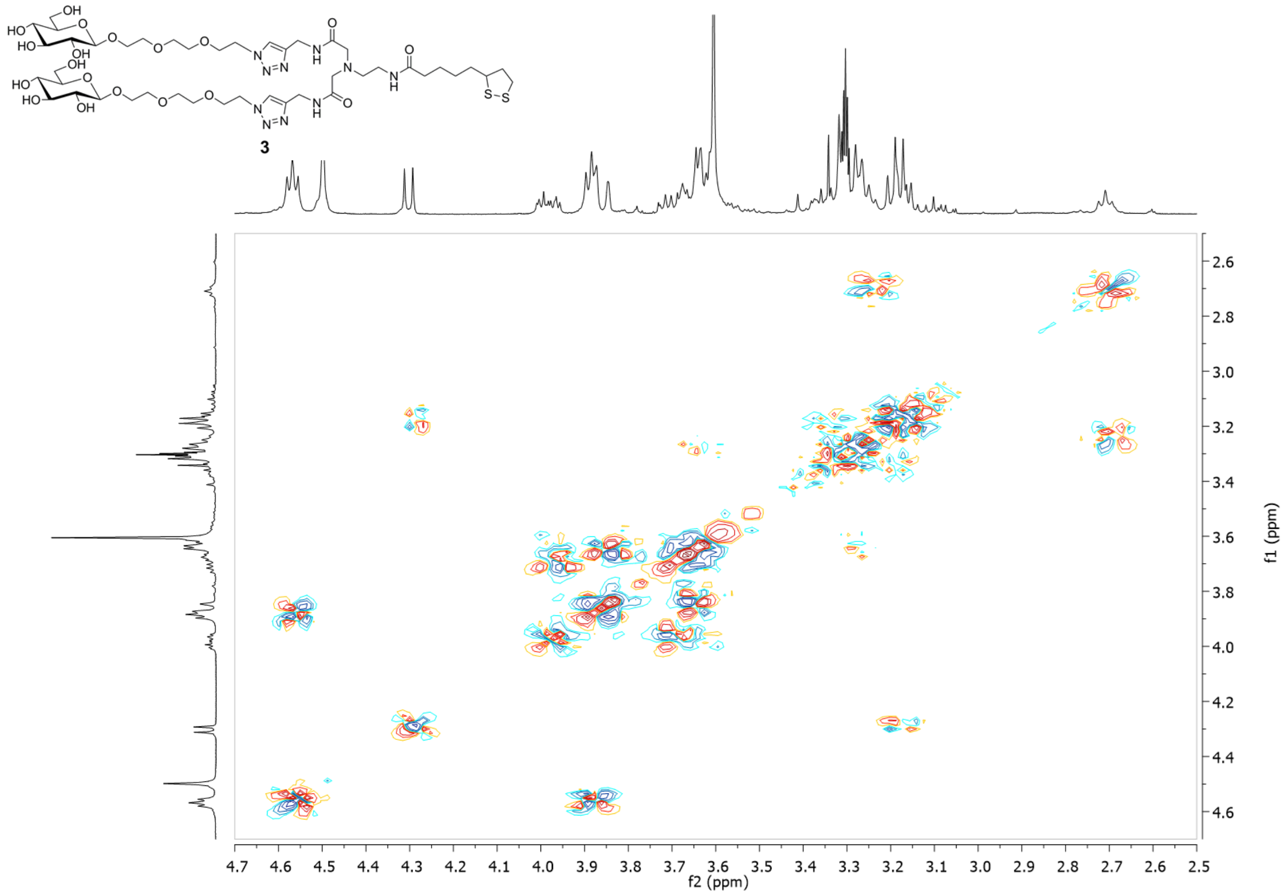

Figure S18. gCOSY-NMR (400 MHz, CD_3OD) of **3.**

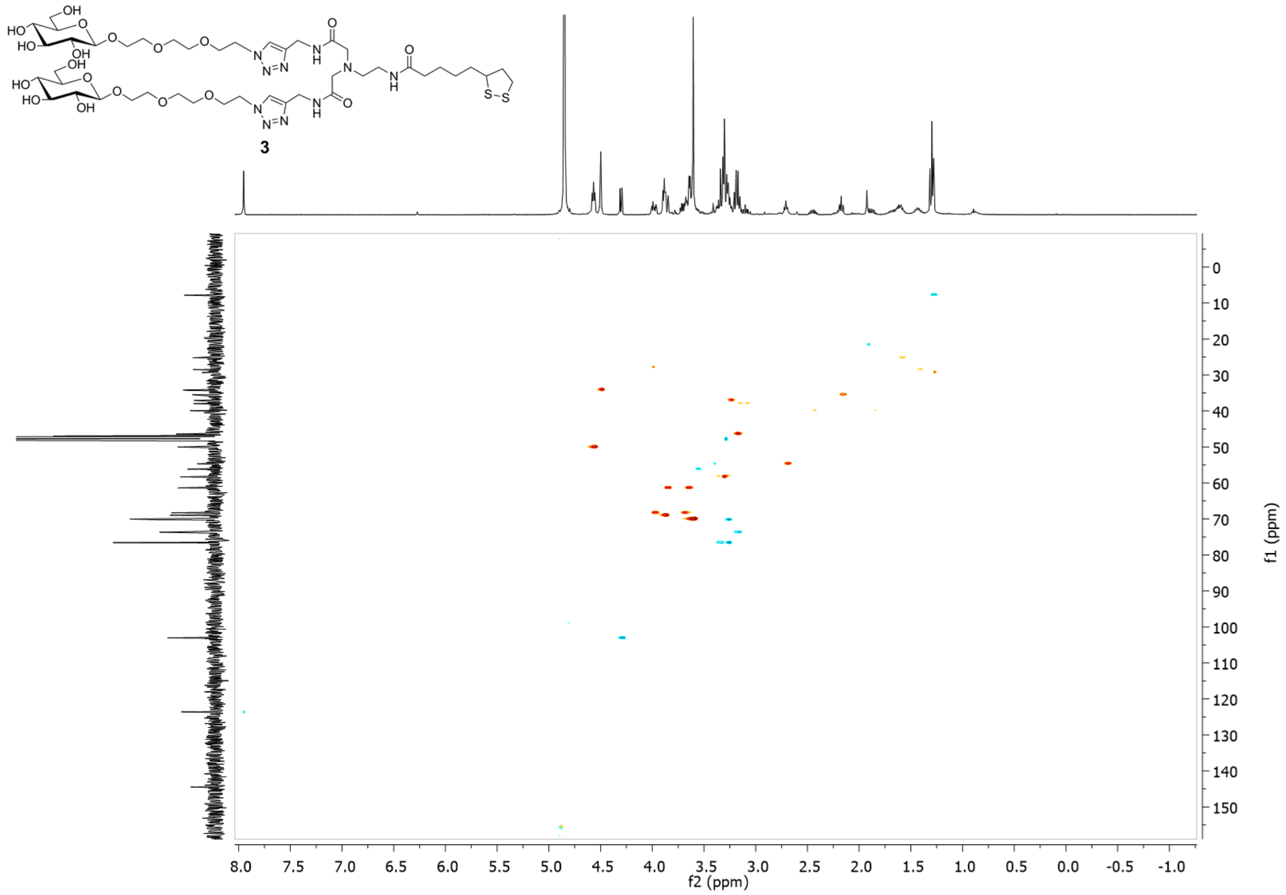

Figure S19. gHSQC-COSY-NMR (400 MHz, CD_3OD) of **3.**

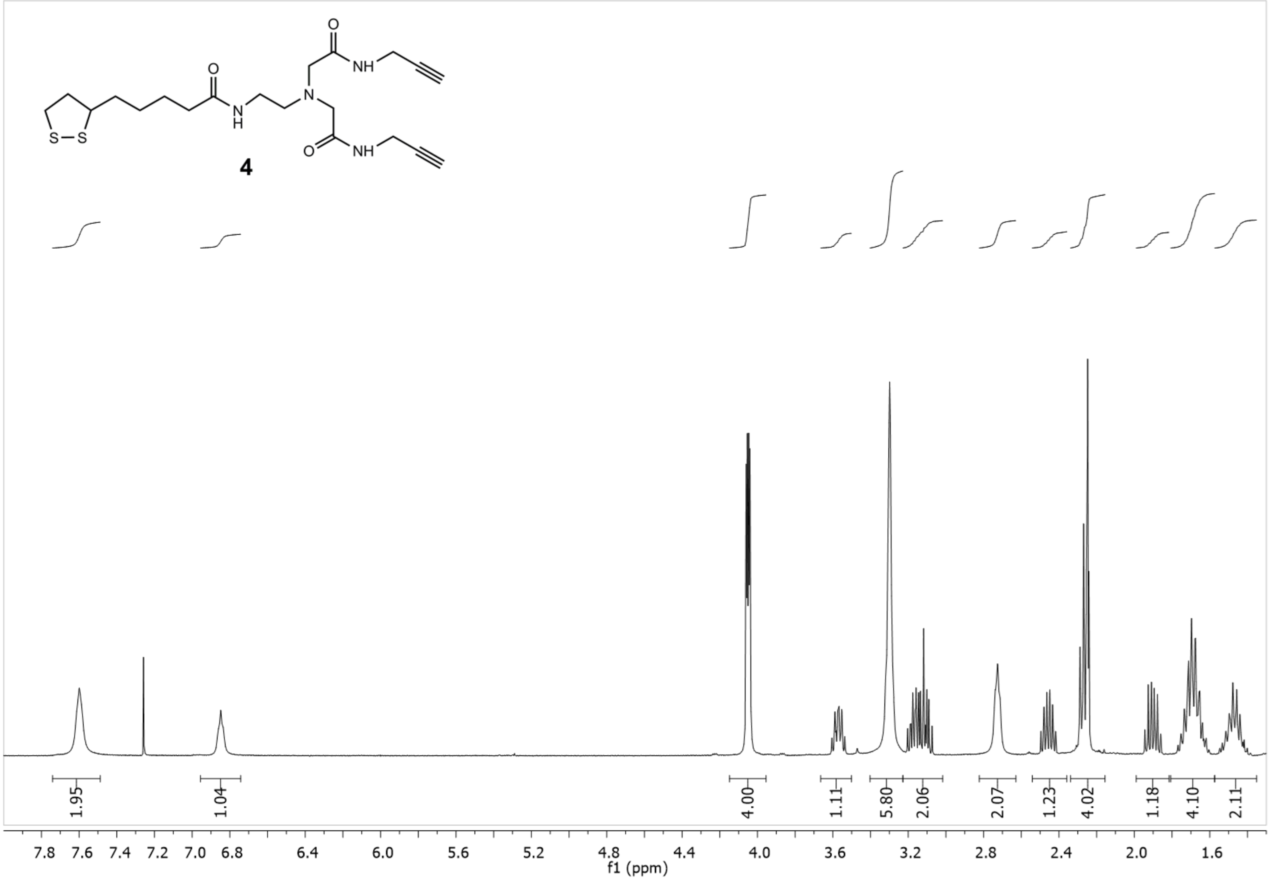

Figure S20. ^{1}H-NMR (400 MHz, $CDCl_3$) of **4.**

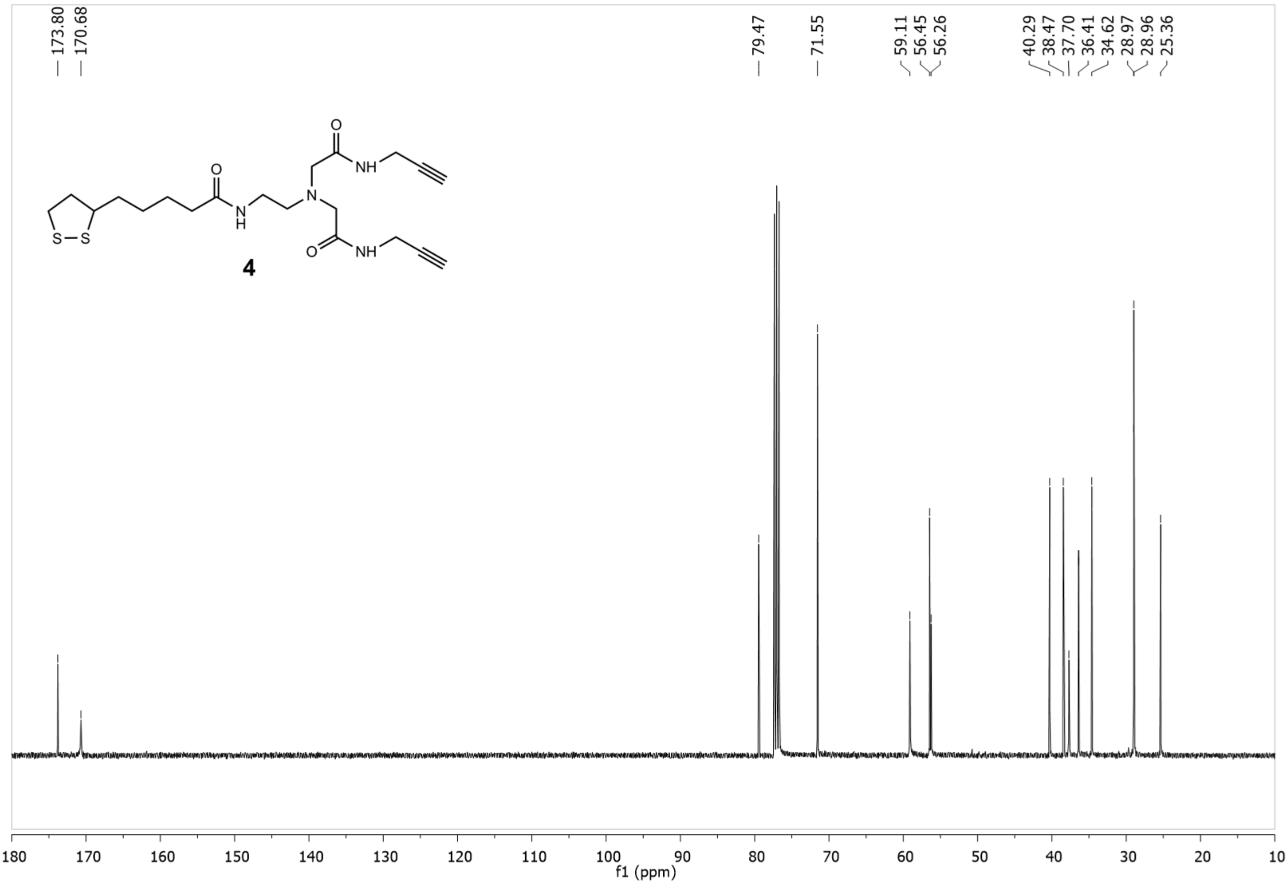

Figure S21. ^{13}C-NMR (100 MHz, $CDCl_3$) of **4.**

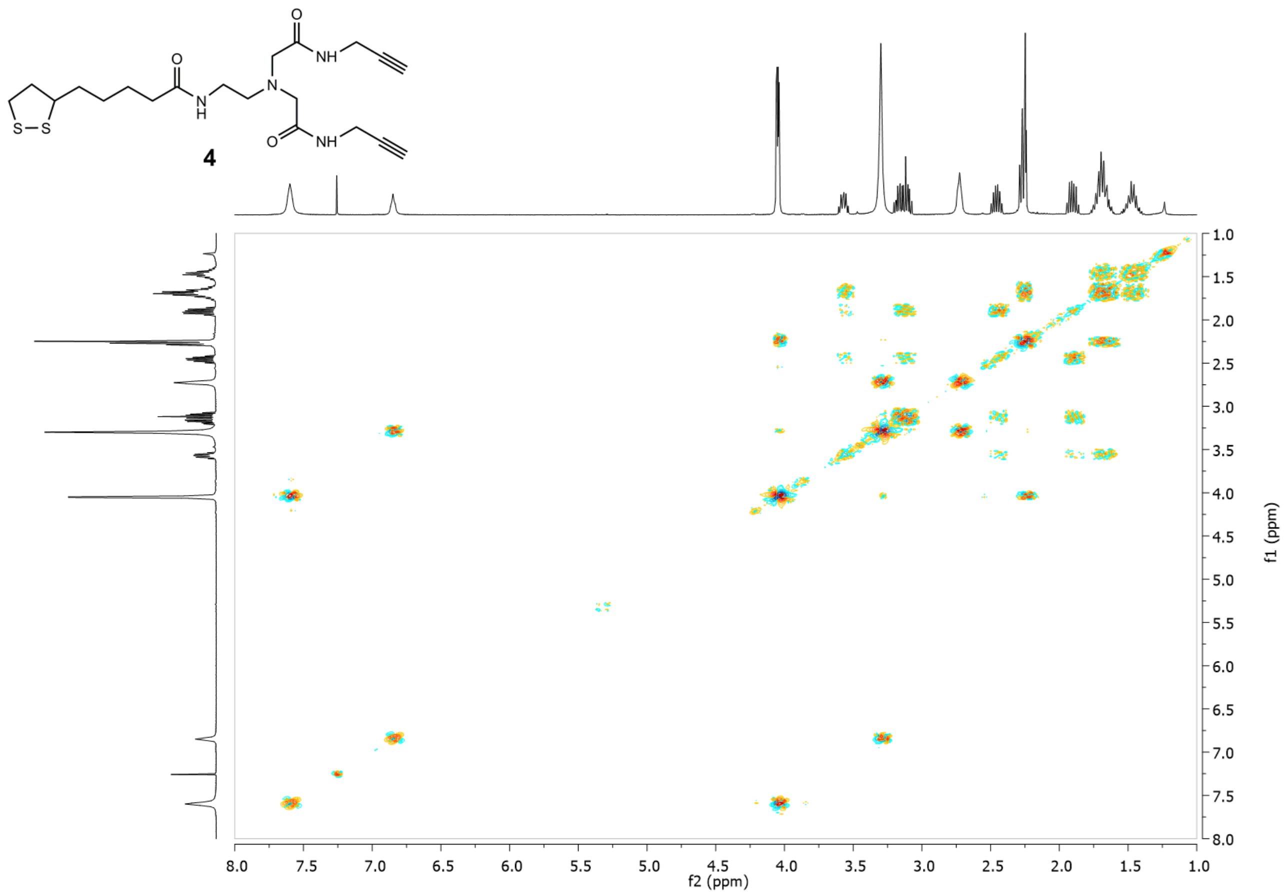

Figure S22. gCOSY-NMR (400 MHz, $CDCl_3$) of **4.**

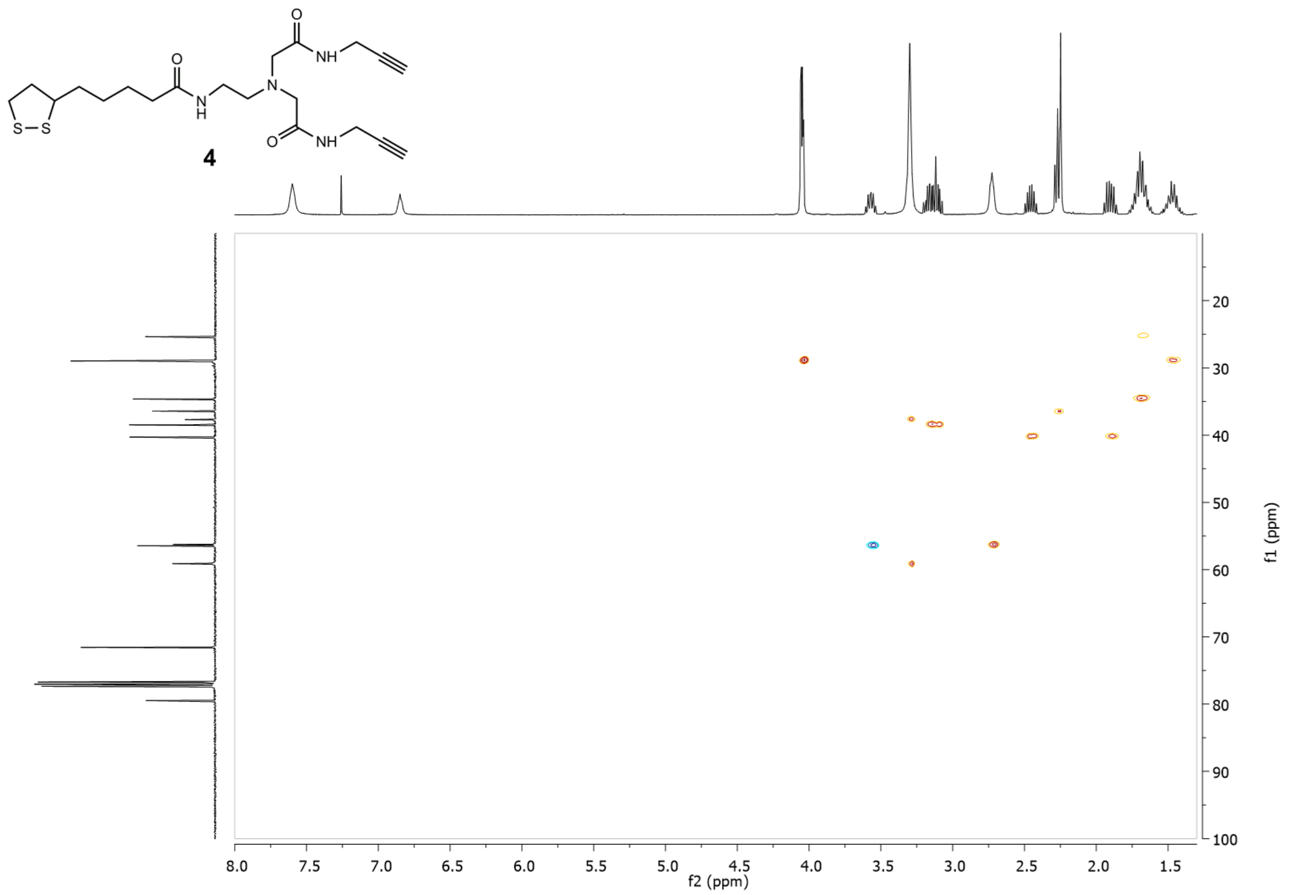

Figure S23. HSQC-NMR (400 MHz, $CDCl_3$) of **4.**

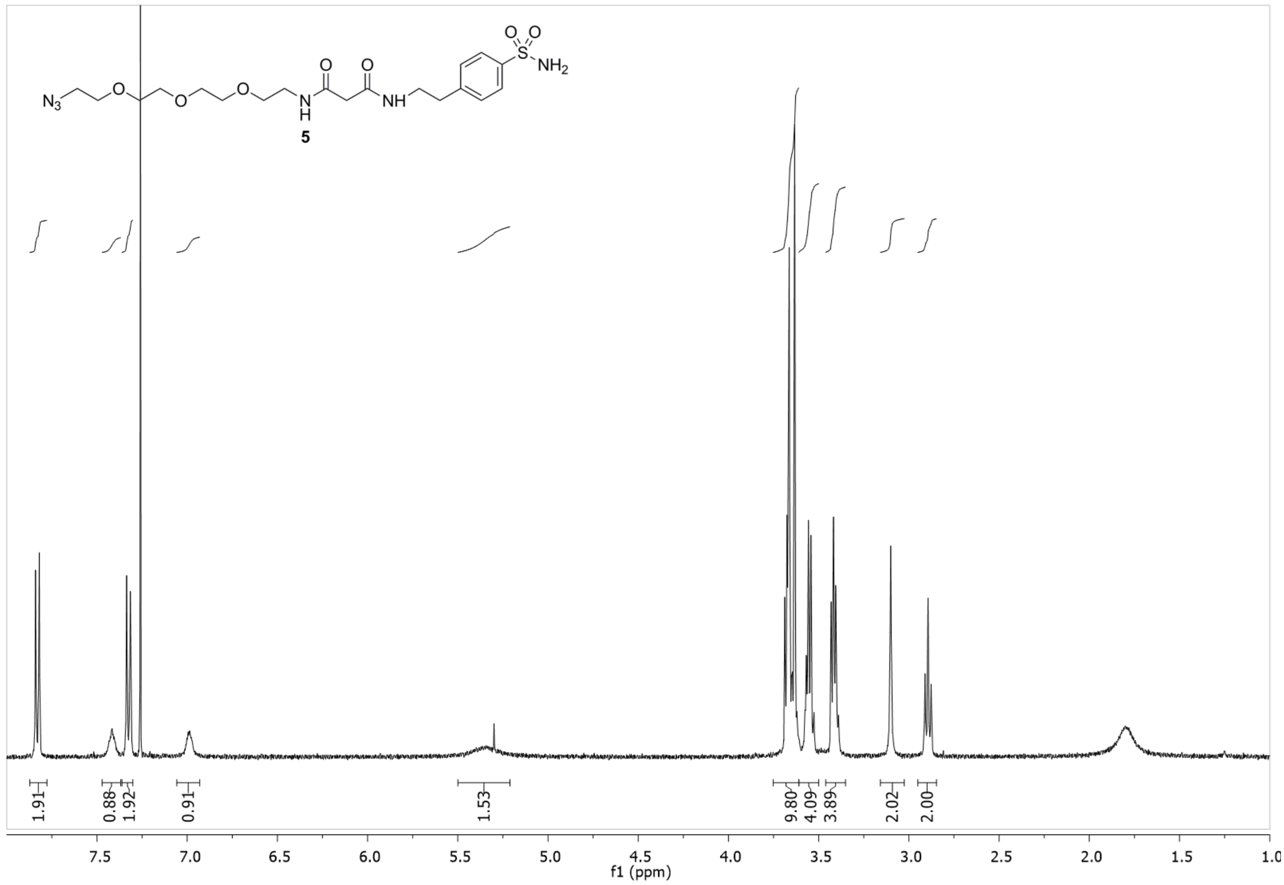

Figure S24. ^{1}H-NMR (400 MHz, $CDCl_3$) of **5.**

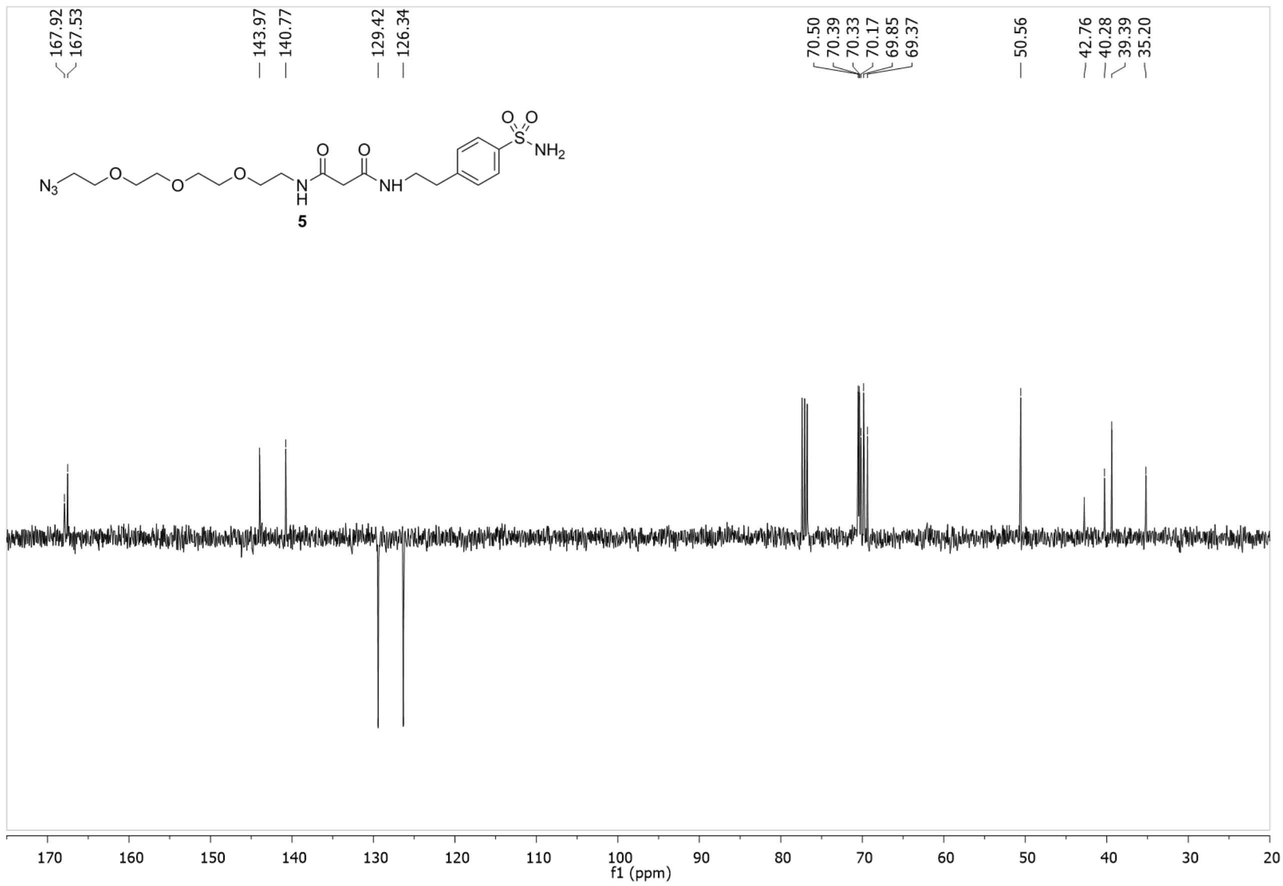

Figure S25. APT-NMR (100 MHz, $CDCl_3$) of **5.**

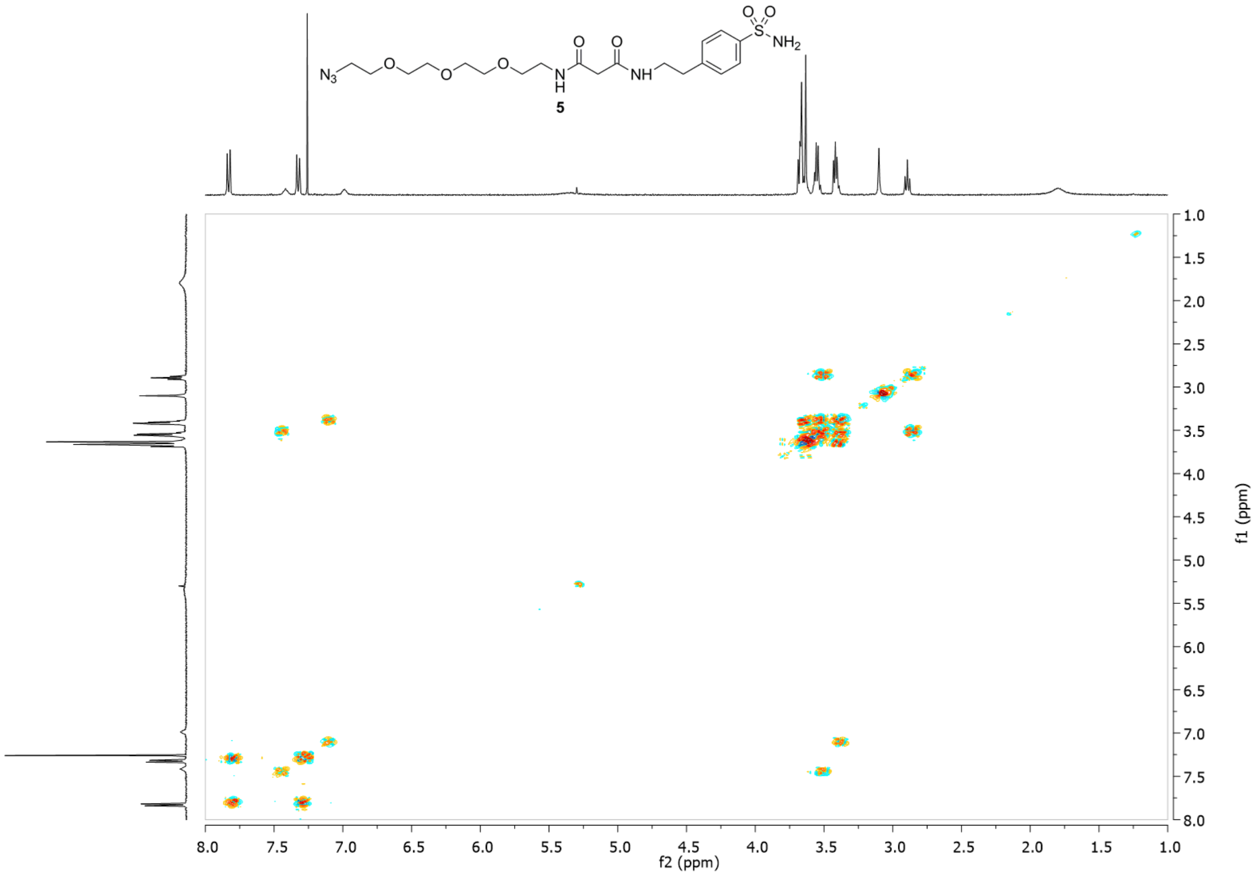

Figure S26. gCOSY-NMR (400 MHz, $CDCl_3$) of **5.**

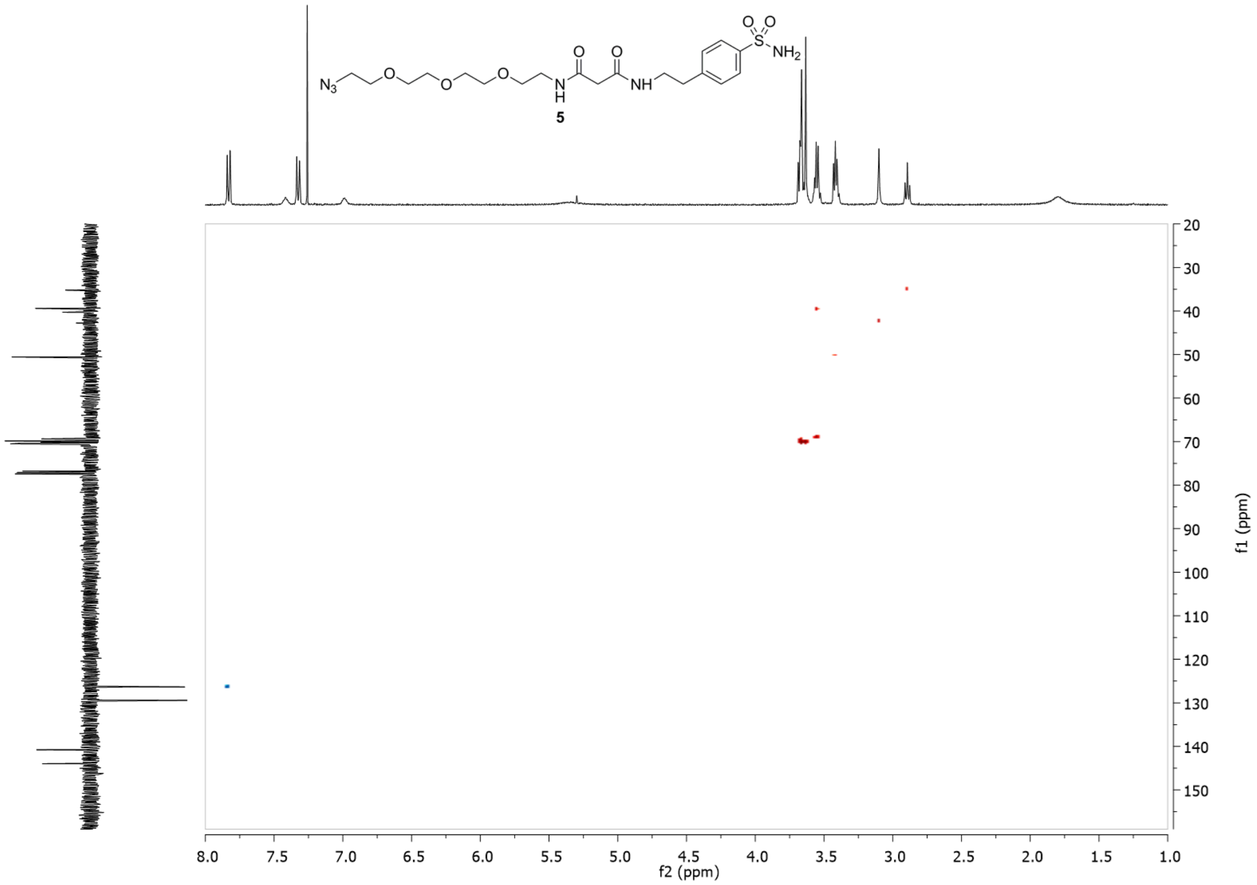

Figure S27. HSQC-NMR (400 MHz, $CDCl_3$) of **5.**

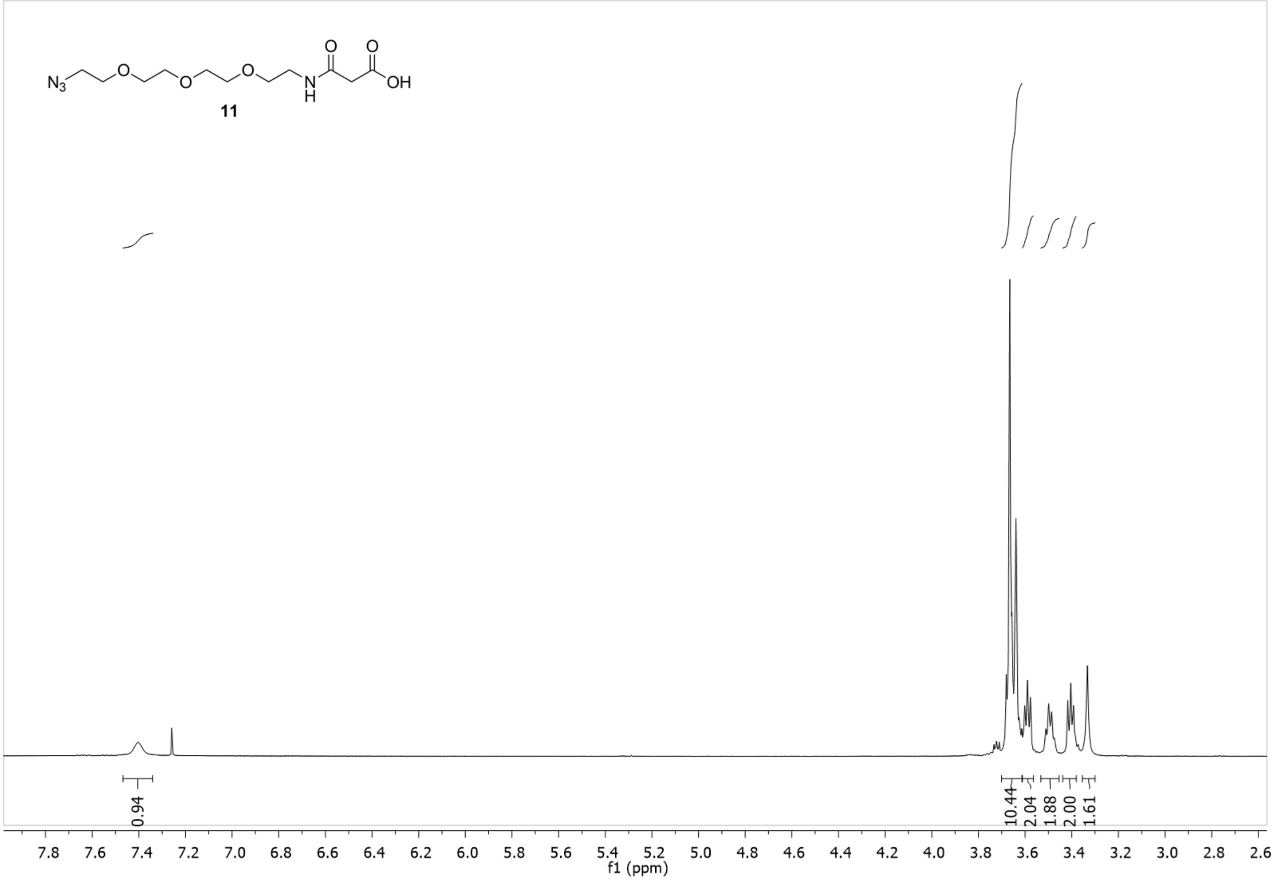

Figure S28. ^{1}H-NMR (400 MHz, $CDCl_3$) of **11.**

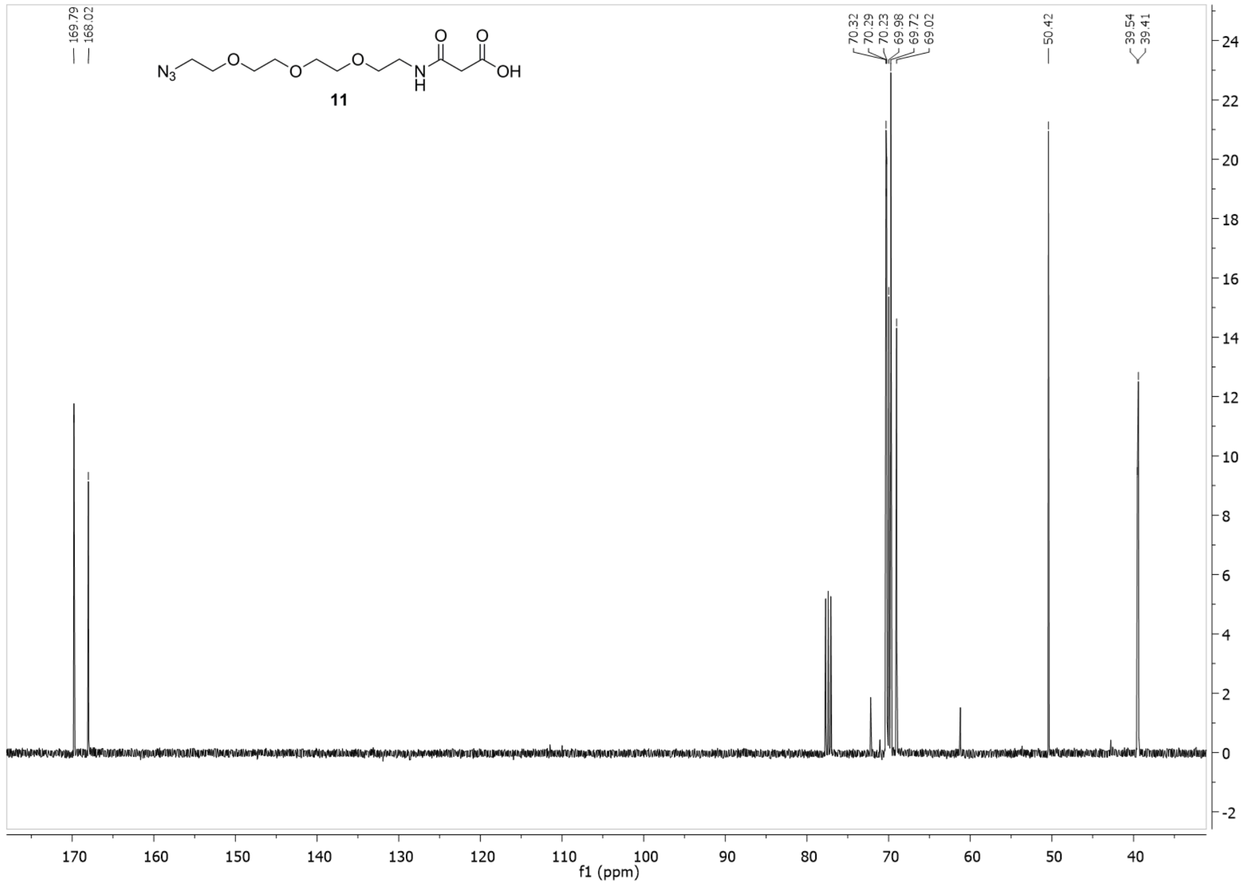

Figure S29. ^{13}C-NMR (100 MHz, $CDCl_3$) of **11.**

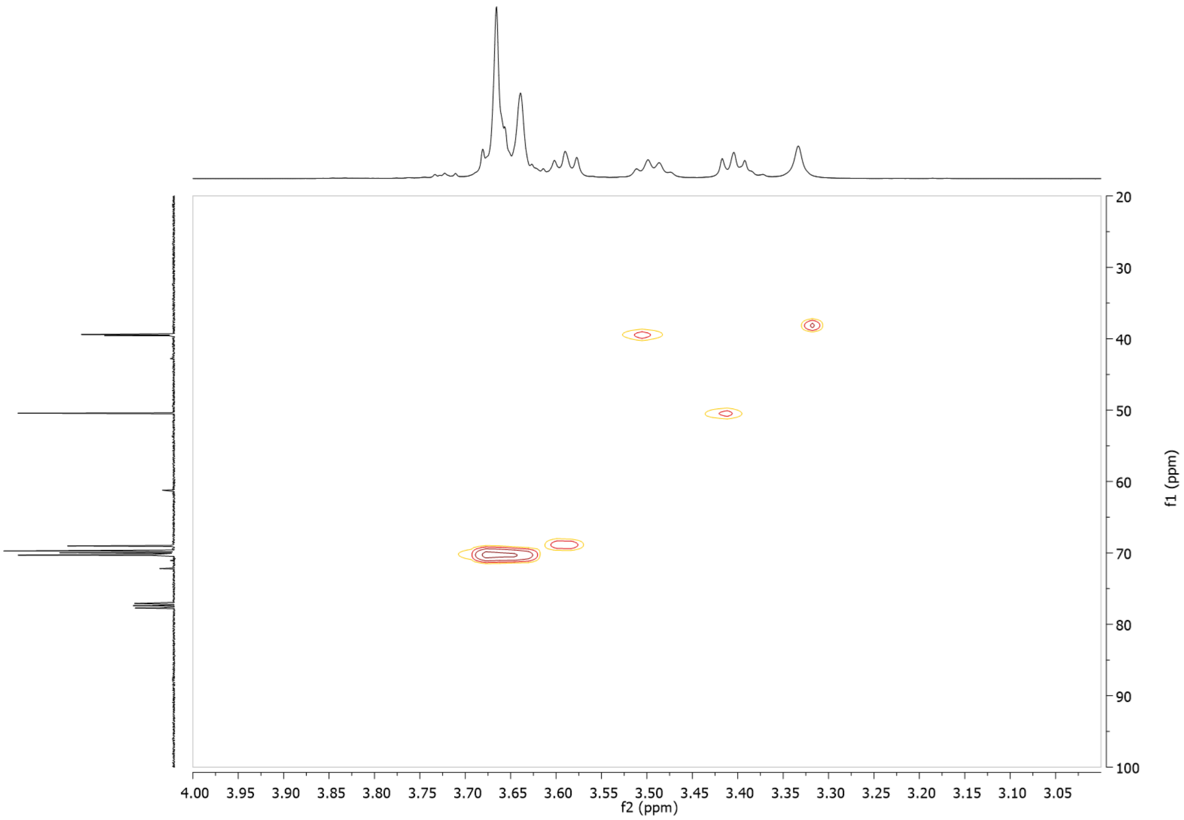

Figure S30. HSQC-NMR (400 MHz, $CDCl_3$) of **11.**

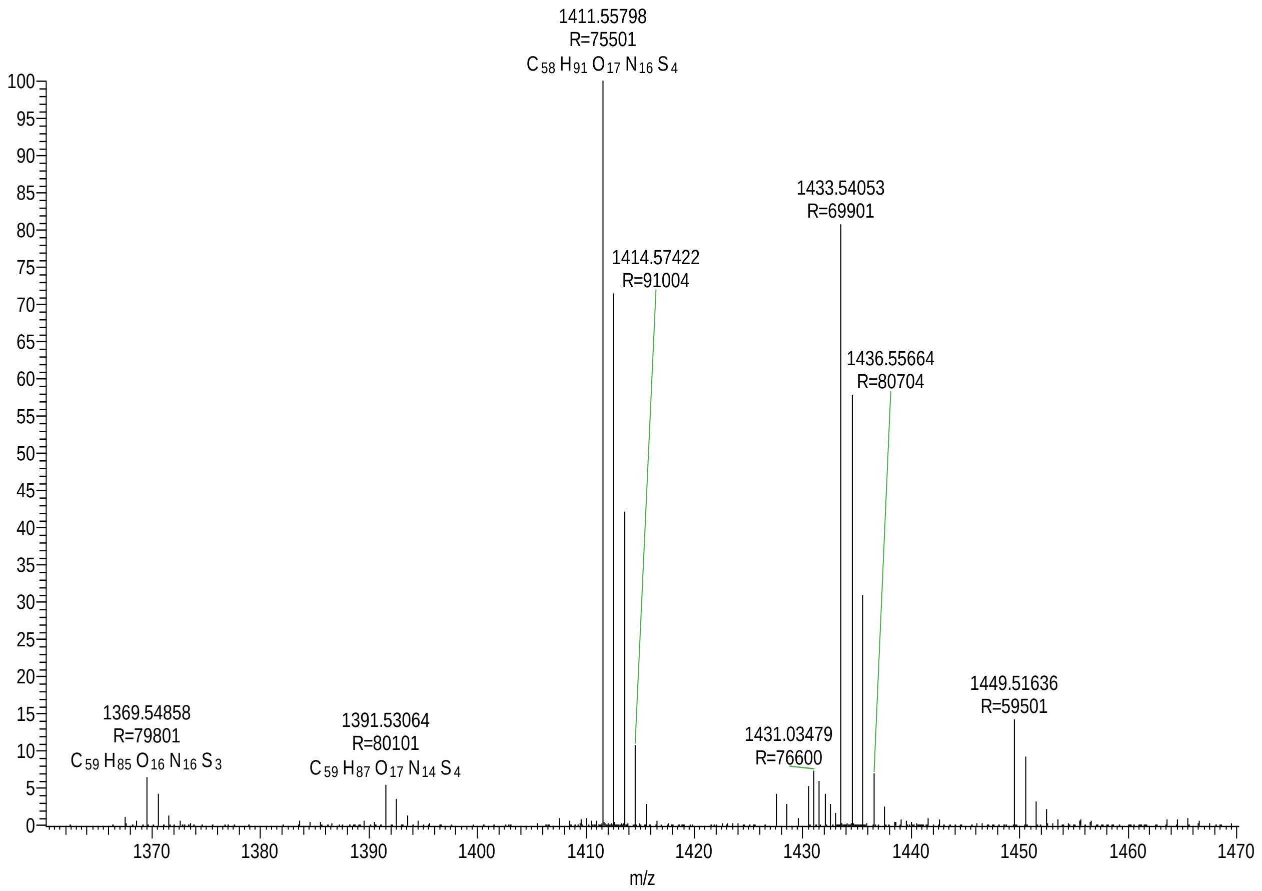

Figure S31. HRMS-ESI (m/z) positive ionization of compound **2**.

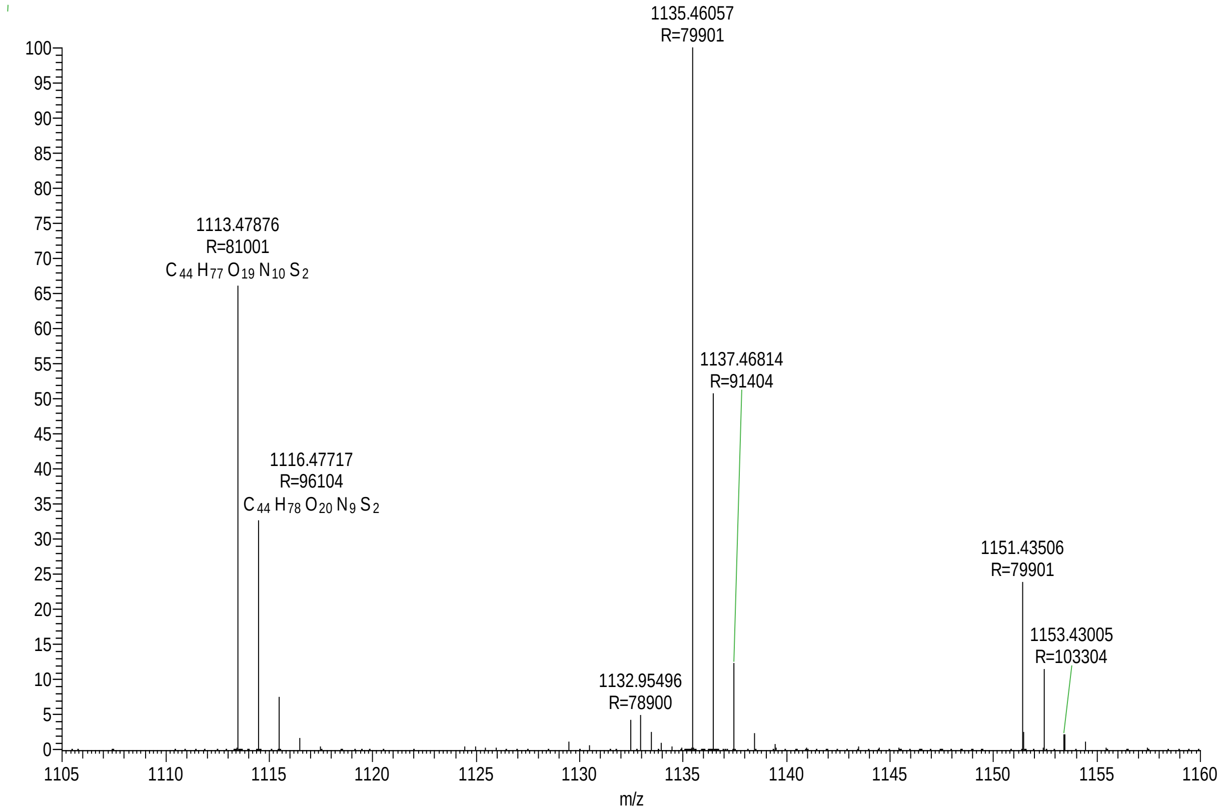

Figure S32. HRMS-ESI (m/z) positive ionization of compound **3**.

References

[1] M. Kluge, B. Schneider, D. Sicker, *Carbohydr. Res.* **1997**, *298*, 147.

[2] W. Shi, S. Dolai, S. Averick, S. S. Fernando, J. A. Saltos, W. L'Amoreaux, P. Banerjee, K. Raja, *Bioconjug. Chem.* **2009**, *20*, 1595.

[3] M. Li, W. Ye, K. Fu, C. zhou, Y. Shi, W. Huang, W. Chen, J. Hu, Z. Jiang, W. Zhou, *Eur. J. Med. Chem.* **2020**, *202*, 112509.

[4] Z. A. Peng, X. Peng, *J. Am. Chem. Soc.* **2002**, *124*, 3343.

[5] S. Mahajan, M. Rani, R. B. Dubey, J. Mahajan, *Int. J. Latest Res. Sci. Technol.* **2013**, *2*, 518.

[6] Z. A. Peng, X. Peng, *J. Am. Chem. Soc.* **2001**, *123*, 183.

[7] C. B. Murray, D. J. Norris, M. G. Bawendi, *J. Am. Chem. Soc.* **1993**, *115*, 8706.

[8] G. Salerno, S. Scarano, M. Mamusa, M. Consumi, S. Giuntini, A. Macagnano, S. Nativi, M. Fragai, M. Minunni, D. Berti, A. Magnani, C. Nativi, B. Richichi, *Nanoscale* **2018**, *10*, 19720.

[9] W. W. Yu, L. Qu, W. Guo, X. Peng, *Chem. Mater.* **2003**, *15*, 2854.

[10] D. Mutavdžić, J. Xu, G. Thakur, R. Triulzi, S. Kasas, M. Jeremić, R. Leblanc, K. Radotić, *Analyst* **2011**, *136*, 2391.
